# Spatiotemporal dynamics of the archaeal community in coastal sediments: assembly process and co-occurrence relationship

**DOI:** 10.1038/s41396-020-0621-7

**Published:** 2020-03-04

**Authors:** Jiwen Liu, Shangqing Zhu, Xiaoyue Liu, Peng Yao, Tiantian Ge, Xiao-Hua Zhang

**Affiliations:** 1 MOE Key Laboratory of Marine Genetics and Breeding, College of Marine Life Sciences, Ocean University of China, Qingdao, 266003 China; 2 Laboratory for Marine Ecology and Environmental Science, Qingdao National Laboratory for Marine Science and Technology, Qingdao, 266071 China; 3 Institute of Evolution & Marine Biodiversity, Ocean University of China, Qingdao, 266003 China; 4 Key Laboratory of Marine Chemistry Theory and Technology, Ministry of Education/Institute for Advanced Ocean Studies, Ocean University of China, Qingdao, 266100 China; 5 College of Chemistry and Chemical Engineering, Ocean University of China, Qingdao, 266100 China

**Keywords:** Microbial ecology, Microbial ecology

## Abstract

Studies of marine benthic archaeal communities are updating our view of their taxonomic composition and metabolic versatility. However, large knowledge gaps remain with regard to community assembly processes and inter taxa associations. Here, using 16S rRNA gene amplicon sequencing and qPCR, we investigated the spatiotemporal dynamics, assembly processes, and co-occurrence relationships of the archaeal community in 58 surface sediment samples collected in both summer and winter from across ~1500 km of the eastern Chinese marginal seas. Clear patterns in spatiotemporal dynamics in the archaeal community structure were observed, with a more pronounced spatial rather than seasonal variation. Accompanying the geographic variation was a significant distance-decay pattern with varying contributions from different archaeal clades, determined by their relative abundance. In both seasons, dispersal limitation was the most important process, explaining ~40% of the community variation, followed by homogeneous selection and ecological drift, that made an approximately equal contribution (~30%). This meant that stochasticity rather than determinism had a greater impact on the archaeal community assembly. Furthermore, we observed seasonality in archaeal co-occurrence patterns: closer inter-taxa connections in winter than in summer, and unmatched geographic patterns between community composition and co-occurrence relationship. These results demonstrate that the benthic archaeal community was assembled under a seasonal-consistent mechanism but the co-occurrence relationships changed over the seasons, indicating complex archaeal dynamic patterns in coastal sediments of the eastern Chinese marginal seas.

## Introduction

Marine sediments are thought to contain approximately half (>10^29^) of the microbial cells in the oceans [1]. Many of these cells are archaea, which may constitute up to 90% of the total prokaryotes in deep subsurface sediments [2]. Recent genome-centric metagenomics and single-cell genomics studies have substantially expanded the known phylogeny and genetic potential of archaea [3–5]. In particular, it has been found that archaea are functionally diverse, being capable of ammonia oxidation, methane metabolism, and organic matter degradation [6–8]. Novel archaeal phyla with unusual metabolic potentials are still being discovered, for example, *Nezhaarchaeota* and *Helarchaeota*, two new archaeal phyla identified in 2019, were found to possess the potential for anaerobic short-chain hydrocarbon cycling [9, 10]. In contrast to the increasing knowledge gleaned from archaeal community composition and metabolic capacity characterization, much less is known about the spatiotemporal dynamics of archaeal community distribution, their assembly mechanism, and inter taxa association in sediments [11, 12]. However, this information is important to gain a better understanding of the ecology of archaea.

Microbial ecology studies using high throughput DNA sequencing have made significant progress in unraveling geographic patterns [13, 14]. The most well-established process is the distance-decay pattern that depicts increasing community dissimilarity with increasing spatial distance [15]. By comparison, the ecological processes that structure microbial distribution patterns are not well understood. Two different processes have been proposed to explain microbial community variations, i.e., deterministic (niche-based) and stochastic (neutral) processes [16, 17]. Increasing efforts are being made to address their relative importance in governing community assembly [18–23]. These two process types have been shown to exert different roles among ecosystems and/or organismal types. For example, stochasticity exerted a stronger effect than determinism on the bacterial community assembly in coastal seawater [24], whereas determinism was more important in lake bacterial communities [25, 26]. Furthermore, bacteria and protists in seawater [27] and bacteria and microbial eukaryotes in lakes [26] have been observed to be differently affected by determinism and stochasticity. These differences raise the question of how the archaeal community, especially that in sediments, is shaped by deterministic and stochastic processes.

The deterministic processes are within the niche theory, which assumes that each taxon has unique and nonoverlapping traits. The impacts of biotic (e.g., competition and predation) and abiotic (e.g., temperature and nutrients) environmental factors are referred to as deterministic processes. In contrast, stochastic processes are within the neutral theory, in which all taxa are assumed to be ecologically functionally equivalent and not subject to any environmental influence. Stochastic processes include typically random events, such as probabilistic dispersal, ecological drift, and random speciation and extinction [12, 28, 29]. Stegen et al. [29, 30] proposed a framework that integrates a null model and phylogenetic information to discern different aspects of environmental selection and stochasticity. Using this framework, both Logares et al. [26] and Wu et al. [27] found that environmental selection was more important than dispersal limitation in structuring planktonic bacterial communities. By contrast, Wang et al. [31] reported that drift was the most important process in the community assembly of archaea in coastal seawater. Differences in spatial scale and environmental gradient among studies might be possible explanations for such disparities. However, little is known about the relative importance of different ecological processes in governing the assembly of archaeal communities in marine sediments [11]. Such knowledge is required for a comprehensive understanding of the community assembly of marine microorganisms.

Interactions between microbes are also important aspects in maintaining a diverse microbial community [32]. Correlation-based network analysis has been extensively utilized to infer microbial interactions, given the difficulty in obtaining microbial pure isolates and co-cultures. The co-occurrence patterns illustrated by a network capture important information in microbial ecology, although they do not necessarily reflect true interactions [12]. However, it remains unclear whether microbial co-occurrence relationships exhibit geographic patterns [33] and whether their dynamics are associated with variations in community composition.

Coastal seas, which are transitional zones between the land and open ocean, are hotspots for biogeochemical cycling. The coastal sediment that receives the sinking particles from the upper waters is a main locus of organic carbon burial [34]. Sedimentary archaea actively process organic carbon and affect the fate of buried organic carbon [8]. The eastern Chinese marginal seas are typical temperate coastal regions; they include the Bohai Sea (BS), Yellow Sea (YS), and East China Sea (ECS). These areas are characterized by clear seasonal variations in environmental conditions, such as water temperature, chlorophyll *a,* and total suspended matter. For example, the seawater in winter is typically colder, more turbid but less productive than in summer [35]. Due to increased anthropogenic activity, many areas of the BS and Changjiang (Yangtze) Estuary (CE) are subject to extensive seasonal hypoxia in the bottom water [36, 37]. In addition, discharges from the Changjiang (Yangtze) River and Yellow River result in relatively high sedimentation rates of up to 42.9 mm/year in mud sediments [38], facilitating the formation of several sedimentary patches that harbor distinct microbial communities [39]. These coastal sediment environments are thus ideal places to test the balance of different assembly processes. It is hypothesized that stochasticity (especially dispersal limitation) is likely to be the dominant process structuring benthic microbial communities, regardless of season, due to the overwhelming effect of differences in sediment source relative to the effect of seasonal environmental changes.

In this study, we examined the geographic pattern of the archaeal community in the eastern Chinese marginal sea sediments at a high spatial scale spanning two seasons (58 samples collected during five cruises), and evaluated the respective contribution from the dominant clades. Furthermore, we provided a quantitative assessment of the ecological processes governing archaeal community assembly and examined whether changes in community compositions affected their co-occurrence patterns. The results revealed that dispersal limitation, homogeneous selection, and drift jointly shaped the distance-decay pattern of the benthic archaeal community in both summer and winter, but that archaeal co-occurrence relationships exhibited seasonality and different spatial variation patterns compared with community compositions.

## Materials and methods

### Sample collection and environmental characterization

Samples were collected from the eastern Chinese marginal seas, an area characterized by a patchy distribution of mud deposits. The sampling areas covered the BS, north Yellow Sea (NYS), south Yellow Sea (SYS), ECS, and CE. A total of 58 surface sediment samples (0–2 cm deep) were collected. Thirty-eight sites were sampled during three cruises conducted in August–September 2015 and October 2015 (termed summer samples), and a further 20 sites were sampled during two cruises conducted in January and March 2016 (termed winter samples) (Supplementary Fig. S1). The sediment samples were collected with a box corer and subsampled using sterile and luer end-removed syringes. One aliquot of the sediment was stored in liquid nitrogen for microbial analyses and a second was stored at −20 °C for physicochemical analyses. Sediment porewater was extracted using a Rhizon sampler attached to a vacuum tube; the vacuum tube was stored at −20 °C. Bottom water salinity, temperature, and dissolved oxygen were recorded by the conductivity–temperature–depth (CTD) system. Dissolved inorganic nutrients in porewaters were analyzed using an AA3 autoanalyser system. Total organic carbon, total nitrogen, bulk δ^13^C, and particle size in sediments were analyzed following the method of Yao et al. [40].

### DNA extraction, PCR and sequencing

Extraction and purification of total DNA from 0.25 g of sediment samples (wet weight) were carried out using the MOBIO PowerSoil DNA Isolation Kit according to the manufacturer’s protocols. The primer set 344F/915R [39] was used for archaeal 16S rRNA gene amplification. Gene amplification was conducted in a 20-μL reaction system containing 4 μL of FastPfu Buffer (5×), 2 μL of dNTP mix (2.5 mM), 0.8 μL of each primer (5 μM), 0.4 μL of Fastpfu polymerase, 10 ng of template DNA, and 0.2 μL of BSA. The PCR parameters were 95 °C for 3 min, followed by 35 cycles of 95 °C for 30 s, 55 °C for 30 s, and 72 °C for 45 s, with a final extension at 72 °C for 10 min. Triplicate amplifications from each sample were mixed for library preparation. Asymmetric barcode sequences were ligated to the PCR primers before amplification. Adapters were then ligated to the amplicons at both ends during library preparation with the NEXTflex™ Rapid DNA-Seq Kit. Sequencing was performed on the Illumina Miseq PE300 platform at Majorbio Bio-Pharm Technology Co., Ltd., Shanghai, China.

### Quantitative PCR

The abundance of total archaea in each sample was measured by quantitative PCR (qPCR) using the primer set 967F/1060R [41]. For each sample, triplicate amplifications were conducted in a 20-μL reaction system containing 10 μL of SYBR Premix ExTaq II (2×), 0.4 μL of ROX, 0.8 μL of each primer (10 μM), and 2 μL of template DNA. The thermal cycling steps consisted of an initial denaturation at 95 °C for 30 s, 40 cycles of 95 °C for 5 s, 50 °C for 30 s, and 72 °C for 30 s, and a final extension at 72 °C for 5 min. The standard curve was generated with tenfold serially diluted linear plasmids containing a single copy of the archaeal 16S rRNA gene (amplified from a coastal sediment sample collected from the YS). The amplification efficiency was 0.95.

### Sequence denoising, OTU clustering and diversity analysis

Raw reads were trimmed with Trimmomatic [42] to remove those of low quality (<20), short length (<100 bp), with any mismatch to barcodes and a maximum of two mismatches to primers. The high-quality pair-end reads were merged using PEAR [43]. Operational taxonomic units (OTUs) were clustered in UPARSE [44] at a 3% dissimilarity level. Singleton and doubleton OTUs, that may represent sequencing errors, were removed for downstream analyses. OTU taxonomy was assigned against the SILVA database (release 123, http://www.arb-silva.de) using the RDP classifier (version 2.2, http://sourceforge.net/projects/rdp-classifier/) [45]. To equalize sequencing depth, each sample was rarefied to 12630 reads (the lowest sequence number across all samples). The rarefied sequences were calculated for Good’s coverage (an estimator of sampling completeness: percentage of the total species is represented in a sample) and alpha diversity indices in Qiime [46]. For beta diversity, the non-metric multidimensional scaling analysis (NMDS) and permutational multivariate analysis of variance (PERMANOVA) were performed based on Bray–Curtis dissimilarities. These analyses were conducted with the “vegan” package in R [47].

The raw reads have been deposited in the NCBI SRA database under the accession number PRJNA507687.

### Distance-decay relationship

Pairwise geographic distances between samples were calculated from the latitude and longitude coordinates using the “geosphere” library, and were plotted against the pairwise Bray–Curtis dissimilarities using the “ggplot2” package in R. A Spearman’s rank correlation between Bray–Curtis dissimilarities and geographic distances was calculated. OTUs belonging to different archaeal groups were also extracted to examine their respective distance-decay relationships.

### Null model, variation partitioning analysis, and partial Mantel test

The framework developed by Stegan et al. [29, 30], that integrates both the phylogenetic and null model analyses, was used to determine the contribution of different ecological processes to community assembly. Using this framework requires significant phylogenetic signal in species niches (i.e., phylogenetic distances among taxa approximate their ecological niche differences) [29]. The phylogenetic signal was evaluated using a Mantel correlogram following the procedure described previously [29, 48]. The inputs used to analyze phylogenetic signal were two distance matrices showing between-OTU differences in environmental optima and between-OTU phylogenetic distances, respectively. All OTUs were included in this analysis. To evaluate ecological processes, β-mean nearest taxon distance (βMNTD), that measures the phylogenetic turnover between samples, was calculated using the R “picante” library. A standardized estimate of βMNTD, i.e., β-nearest taxon index (βNTI), was calculated as the number of standard deviations of the observed βMNTD from the null distribution of βMNTD. βNTI values >2 or <−2 indicate heterogeneous (significantly more than expected phylogenetic turnover) and homogeneous (significantly less than expected phylogenetic turnover) selection, respectively, which represent the deterministic process. βNTI values falling within the range of −2 to 2 (do not significantly deviate from the null βMNTD distribution) indicate stochastic processes that include homogenizing dispersal (mass effect), dispersal limitation, and drift. To discern these three processes, a Bray-Curtis based Raup–Crick metric (RC_bray_) was calculated with RC_bray_ > 0.95, |RC_bray_| < 0.95 and RC_bray_ < −0.95 being interpreted as dispersal limitation, drift, and homogenizing dispersal, respectively.

Variation partitioning analysis (VPA) was conducted to address the relative role of environmental and spatial factors and their combined effect. Environmental factors that were significant in explaining community variations in the canonical correlation analysis (CCA) were used for further analysis. A set of spatial factors was generated using the principal coordinates of neighbor matrices (PCNM) analysis and were selected in the same way as environmental factors for the VPA analysis. The pure effects of environmental (E|S) and spatial (S|E) factors were tested for significance using the permutation test. The partial Mantel test was conducted to verify the results obtained from VPA. Collinearity between environmental variables was detected and environmental variables with a variance inflation factor >10 were removed in downstream analyses. All these analyses were performed in the R software.

### Network analysis

Co-occurrence networks were constructed using the “igraph”, “Hmisc” and “qvalue” libraries in R. To reduce complexity, only OTUs with a proportion above 0.01% across all samples and occurring in more than 20% of all samples were retained. The pairwise Spearman’s correlations between OTUs were calculated, with a correlation coefficient > |0.7| and a *P* value < 0.01 (Benjamini and Hochberg adjusted) being considered as a valid relationship. The network-level (mean node degree, clustering coefficient, average path length, modularity, density, diameter, betweenness centralization and degree centralization) and node-level (degree, transitivity, betweenness centrality, and closeness centrality) topological features of a network were calculated. In addition, the subgraph of each sample was extracted from the meta-community network to examine the distance-decay relationship of the co-occurrence patterns. The network was visualized in Gephi (https://gephi.org).

## Results

### Archaeal abundance, diversity, and community composition

**Fig. 1:**
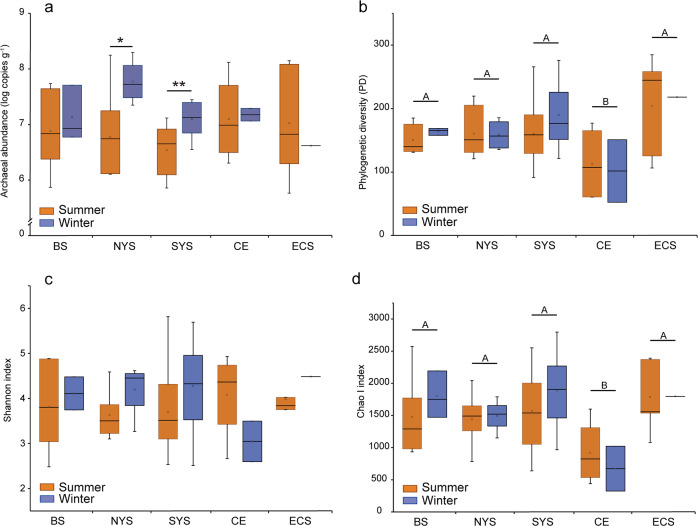
The archaeal abundance and diversity in sediments of the eastern Chinese marginal seas. 16S rRNA gene copy numbers derived from qPCR (**a**) and indices of alpha diversity shown as phylogenetic diversity (**b**), Shannon (**c**) and Chao I (**d**). Significant differences between seasons in each sampling area are marked by stars (**P < 0.01; *P < 0.05). Significant differences between sampling areas are marked by different capital letters. BS, Bohai Sea; NYS, north Yellow Sea; SYS, south Yellow Sea; ECS, East China Sea; CE, Changjiang Estuary.

The copy numbers of the archaeal 16S rRNA gene varied from 5.80 × 10^5^ (P03 of ECS in summer) to 1.97 × 10^8^ copies g^−1^ (B27 of NYS in winter) across all samples. The archaeal abundance showed no significant variations across sampling areas, although appeared to be lowest at SYS in summer (Fig. 1a). In contrast, there were clear seasonal differences in archaeal 16S rRNA gene abundance across all samples (*P* < 0.01, Wilcoxon rank-sum test), with higher values observed in winter (2.9 ± 4.3 × 10^7^ copies g^−1^) compared with summer (2.2 ± 4.4 × 10^7^ copies g^−1^). This winter increase in archaeal abundance was more pronounced in NYS and SYS compared with other areas (Fig. 1a). Furthermore, there was a greater level of intra-area variation in archaeal abundance in summer (standard deviations of 0.44–0.95 across areas) compared to winter (standard deviations of 0.11–0.40 across areas; Fig. 1a) samples, which may suggest niche partitioning within a small spatial scale in summer.

A total of 732,540 reads were obtained after quality control and rarefication, and these were clustered into 7979 OTUs at a 97% similarity level. The Good’s coverage values varied between 93.6 and 99.4% across samples (Supplementary Table S1), in line with the species accumulation curve that approached an asymptote (Supplementary Fig. S2), suggesting that the sequencing effort recovered most of the local species diversity. There was no significant difference in alpha diversity amongst the sampling areas, except for CE. The CE samples were found to have the lowest level of species richness and phylogenetic divergence regardless of season, as evidenced by the significantly lower numbers of OTUs (Supplementary Table S1), phylogenetic diversity (PD), and Chao I (Fig. 1b, d) indices compared with other areas. However, the Shannon index revealed a similar species diversity between CE and others areas in summer (Fig. 1c). In BS and YS, the average values of Shannon, PD, and Chao I indices appeared to be higher in winter than in summer samples (Fig. 1b–d) but the comparisons were not statistically significant.

**Fig. 2:**
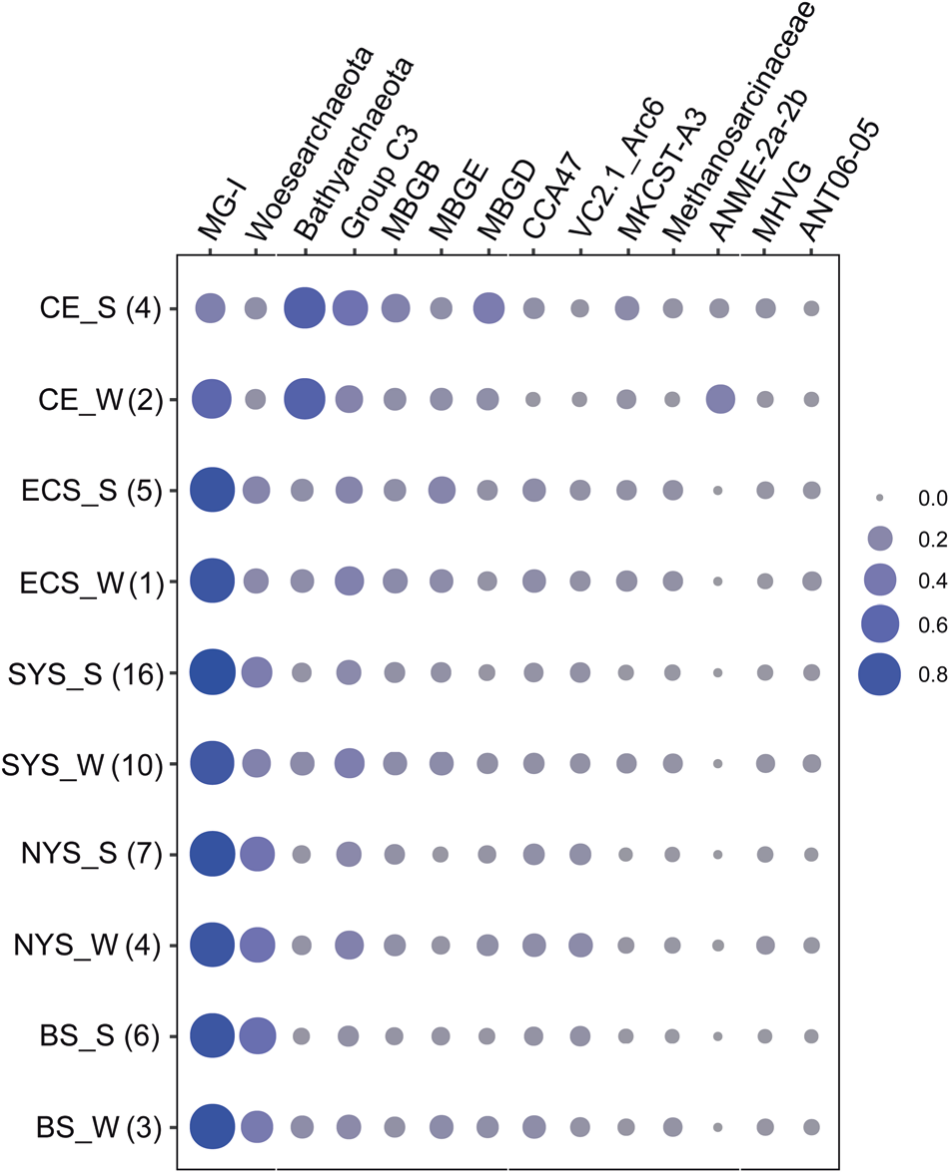
The composition of dominant archaeal clades in sediments of the eastern Chinese marginal seas. Samples from the same area and same season are combined and numbers in parenthesis indicate the number of samples for each group. Group names are defined by area and season (S, summer; W, winter). The relative abundances were squarely transformed. MG-I Marine Group I, MBGB Marine Benthic Group B, MBGD Marine Benthic Group D, MBGE Marine Benthic Group E, MHVG Marine Hydrothermal Vent Group.

Taxonomic assignment revealed that an average of 90.7 ± 10.8% of sequences in each sample could be classified. The sediment samples were dominated by the TACK superphylum (73.5 ± 15.8%), followed by DPANN (10.7 ± 12.0%), *Euryarchaeota* (5.5 ± 6.5%) and Asgard archaea (1.6 ± 6.5%). A resolved phylogenetic classification showed contrasting community compositions over spatial and seasonal scales (Fig. 2). Spatially, Marine Group I (MG-I) of *Thaumarchaeota* (62.3 ± 25.5%) and *Woesearchaeota* (formerly known as DHVEG-6, 10.5 ± 12.0%) were the two most abundant archaeal clades (Supplementary Fig. S3). These two clades were more abundant in BS, YS and ECS compared to CE (*P* < 0.01). MG-I was evenly distributed in BS, YS, and ECS, whereas *Woesearchaeota* was more abundant in BS and NYS compared with SYS and ECS (*P* < 0.01). Comparatively, the CE samples were mainly populated by *Bathyarchaeota*, which comprised 43.2 ± 30.7% of the CE community. Also, anaerobic methanotrophic archaea increased in relative abundance in CE samples compared with those from other areas. Seasonally, MG-I and *Woesearchaeota* were consistently dominant in both summer and winter samples, whereas Group C3 was more abundant in winter than in summer at SYS (*P* < 0.01).

### OTU occurrence pattern

**Fig. 3:**
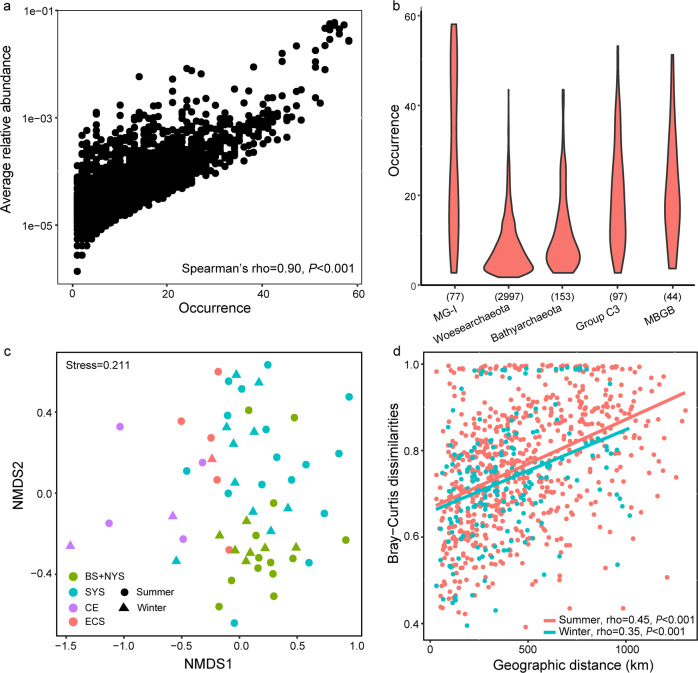
The site occurrence pattern of OTUs and OTU-level community comparison and distance-decay pattern. **a** Abundance–occupancy relationship based on all OTUs. Spearman’s rank correlation was calculated between average relative abundance and numbers of site occurred; **b** Occurrence patterns of OTUs belonging to the top five most abundant archaeal clades. Numbers in parenthesis indicate the number of OTUs for each clade; **c** Ordination of community using the non-metric multidimensional scaling based on Bray–Curtis dissimilarities; **d** Pairwise relationships between Bray–Curtis dissimilarities and geographic distances for both winter and summer samples. Spearman’s rank correlations were calculated.

Approximately 2% (167 out of 7979) of OTUs occurred in >50% of all samples, and two OTUs belonging to MG-I were present throughout. There was a significant positive relationship between relative abundance and sites occupied by each OTU (Fig. 3a), indicating that rare taxa tended to have a weak ability to disperse and/or adapt. The occurrence pattern of OTUs affiliated with each dominant archaeal clade was also investigated. *Woesearchaeota* was found to have an extremely high number of OTUs (2997), tenfold higher than other clades. However, most of the *Woesearchaeota* OTUs occurred at fewer than ten sites (Fig. 3b). A similar pattern was seen in the occurrence of *Bathyarchaeota* OTUs. MG-I, although having the highest abundance, showed a low level of diversity constituting of only 77 OTUs. In contrast to *Woesearchaeota* and *Bathyarchaeota*, the MG-I OTUs showed an even pattern of distribution (Fig. 3b).

### Geographic pattern of archaeal community

The NMDS analysis showed a clear separation of communities by sampling site, which was consistent with the variation in relative abundance of dominant archaeal clades across samples. Basically, all the samples were partitioned into four geographic clusters, i.e., BS and NYS, SYS, ECS, and CE (Fig. 3c). This pattern was supported by the PERMANOVA analysis in which significant cluster-to-cluster variations were observed (Supplementary Table S2). Intra-zone community variations seemed to be more apparent in SYS than in BS and NYS. In comparison, there was no significant season-to-season separations according to the PERMANOVA analysis; this was consistent with the NMDS analysis, in which samples from the same season did not cluster together. Overall, the results revealed that the benthic archaeal communities were more variable among spatial areas than between seasons.

**Fig. 4:**
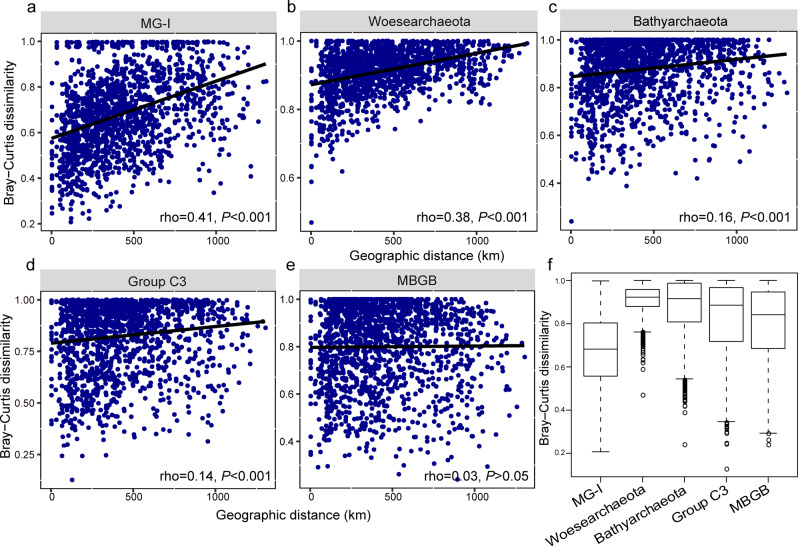
Bray-Curtis dissimilarity patterns of major archaeal clades. Distance-decay patterns of the top five most abundant archaeal clades (rank in relative abundance, **a**–**e**) and their respective OTU-level Bray–Curtis dissimilarities (**f**).

The observation of similar archaeal assemblages in geographically close samples was reflected by a clear distance-decay pattern (increased community dissimilarity with increasing geographic distance) (Fig. 3d). This pattern was seen in both summer (rho = 0.45, *P* < 0.001) and winter (rho = 0.35, *P* < 0.001) samples. To discern the respective contributions of different lineages to the meta-community geographic pattern, the distance-decay relationships of the top ten most abundant archaeal clades, i.e., MG-I, *Woesearchaeota*, *Bathyarchaeota*, Group C3, Marine Benthic Group B, Marine Benthic Group E, Marine Benthic Group D, CCA47, VC2.1_Arc6, and MKCST-A3 (Supplementary Fig. S3) were examined. MG-I, *Woesearchaeota*, *Bathyarchaeota*, Group C3 (Fig. 4a–d), Maine Benthic Group D and MKCST-A3 (Supplementary Fig. S4b and 4e) displayed a significant correlation between community dissimilarity and geographic distance. For the top five most abundant clades, the community dissimilarity-geographic distance correlations decreased gradually with relative abundance, as evidenced by the rho values (Fig. 4a–e). MG-I exhibited the highest correlation, which was indicative of the strongest distance-decay pattern. However, it was interesting that the Bray–Curtis dissimilarities of MG-I across samples were the lowest compared with those of other dominant clades (Fig. 4f). An in-depth phylogenetic analysis was performed to explore whether the strong distance-decay pattern of MG-I was attributed to the habitat preference of different OTUs. The results showed that most of the MG-I OTUs were affiliated with the subclades (alpha I and III) that have mostly been retrieved from the marine environment (Supplementary Fig. S5).

### Ecological processes governing community structure

**Fig. 5:**
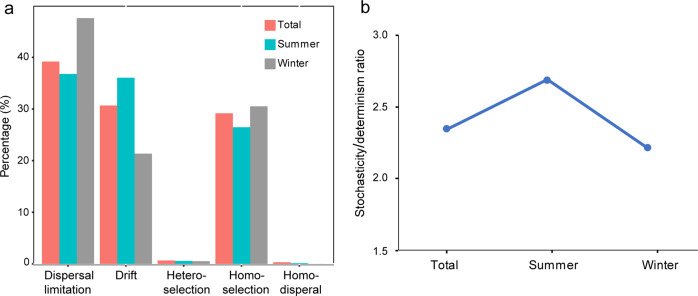
Null model analysis revealing the assembly mechanism of the archaeal community in summer, winter and across all samples. **a** Relative contribution of different ecological processes; **b** The ratio of stochasticity and determinism.

To explore mechanisms underpinning the observed geographic pattern, the relative roles of niche and neutral processes in community assembly were analyzed. Significant phylogenetic signal was found across relatively short phylogenetic distances (Supplementary Fig. S6), indicating that βMNTD was the appropriate distance to measure phylogenetic turnover. The null model showed that dispersal limitation was the most important process, accounting for 39.2% of the community variation across all samples (Fig. 5a). This was followed by drift and homogeneous selection, each of which explained ~30% of the total variation. This balance of different ecological processes was generally conserved in both summer and winter samples, although slight variations were observed. For example, dispersal limitation and drift exerted a greater role in winter and summer, respectively (Fig. 5a). Overall, the results suggested that stochastic processes explained a higher proportion of the archaeal community variation than deterministic processes, and the proportion was slightly higher in summer than in winter (Fig. 5b). This finding was further consolidated by an inference of ecological processes using OTUs defined at a 99% similarity level, which showed a dominance of stochasticity relative to determinism (Supplementary Fig. S7).

**Fig. 6:**
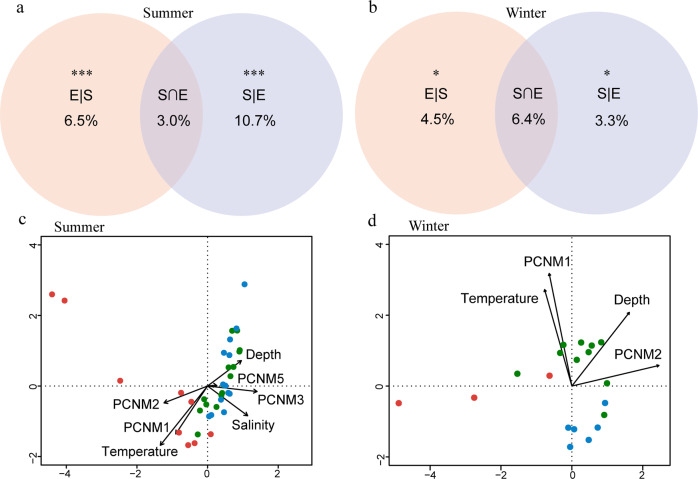
Influence of spatial and environmental factors as shown by the variation partitioning analysis and canonical correlation analysis. Variation partitioning analysis performed to quantify the contribution of spatial and environmental factors to community variations in summer (**a**) and winter (**b**). Canonical correlation analysis conducted to show significant spatial and environmental factors in governing the assembly of summer (**c**) and winter (**d**) archaeal communities. PCNMs, geographic factors generated using principal coordinates of neighbor matrices. Depth indicates water depth of each station. Dot color indicates samples from different areas: red, CE and ECS; green, SYS; blue, BS and NYS.

VPA (Fig. 6a, b) and partial Mantel test (Supplementary Table S3) were performed to verify the results of the null model. Both analyses revealed a higher effect of spatial factors (S|E) than that of environmental factors (E|S) in summer samples. For the winter samples, partial Mantel tests revealed a significantly higher effect of spatial factors, which was in line with the null model analysis, whereas VPA appeared to show a higher effect of environmental factors. The lower robustness among different methods for winter samples, however, suggests that spatial factors play a relatively less important role here. This is consistent with a higher explanatory power of stochasticity relative to determinism in summer than in winter (Fig. 5b). For both seasons, >70% of the community variation could not been explained, which implies complex processes of community assembly. The significant factors were four spatial factors (PCNM 1–3, 5) and three environmental factors (temperature, water depth and salinity; Supplementary Table S4) in summer, and two spatial factors (PCNM 1 and 2) and two environmental factors (temperature and water depth) in winter as shown by the CCA analysis (Fig. 6c, d).

### Archaeal co-occurrence networks

**Fig. 7:**
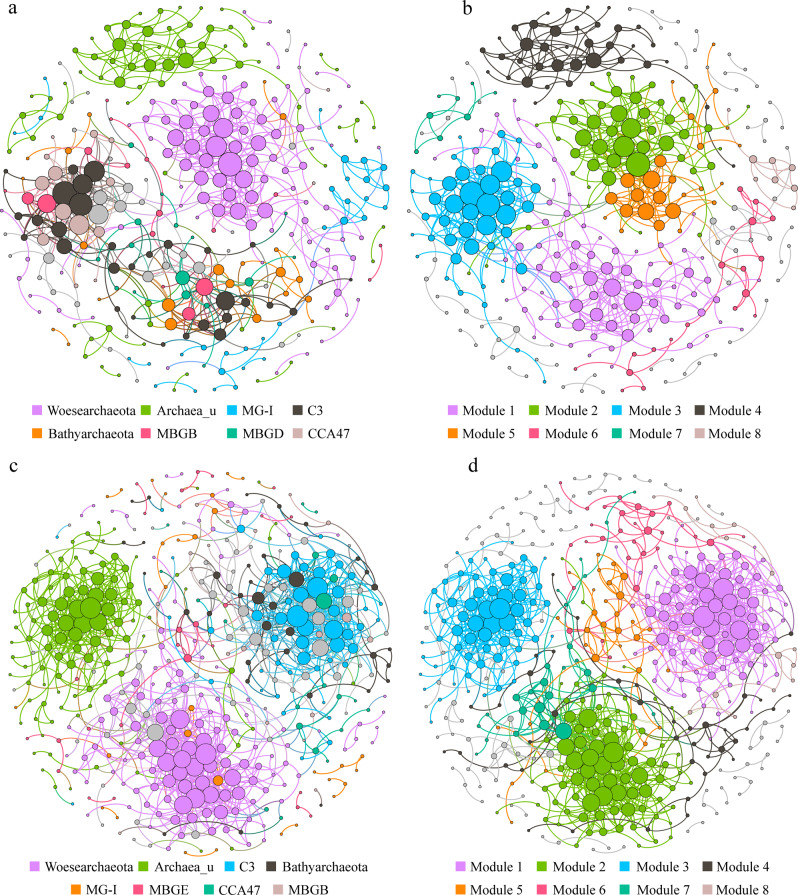
Co-occurrence networks of the archaeal community based on pairwise Spearman’s correlations between OTUs. Each shown connection has a correlation coefficient >|0.7| and a *P* value < 0.01. The size of each node is proportional to the number of connections. The upper panel shows the network of summer samples with OTUs colored by taxonomy (**a**) and modularity (**b**); the lower panel shows the network of winter samples with OTUs colored by taxonomy (**c**) and modularity (**d**).

The correlation-based network consisted of 334 nodes (OTUs) and 690 edges (correlations) for the summer community, and 465 nodes and 1257 edges for the winter community. OTUs belonging to the same archaeal clade were inclined to co-occur with one another (Fig. 7a, c). Modularity analysis revealed eight major modules (subunits with highly inter-connected nodes) in both the summer and winter networks (Fig. 7b, d). Many of these modules were comprised of a group of OTUs that were phylogenetically close and belonged to the same clade. For example, module 2 and 5 in the summer network and module 1 in the winter network were predominated by *Woesearchaeota* OTUs and Group C3 OTUs, respectively. This finding suggests that taxonomic relatedness plays a key role in determining the network modular structure. Additionally, the archaeal co-occurrence pattern varied between seasons. MG-I OTUs were more dispersedly distributed in winter than in summer, whereas an opposite trend was seen for the co-occurrence pattern of Group C3.

Eight parameters that represented the network topological structure were calculated. The modularity, betweenness centralization and diameter appeared to be higher in the summer network compared to the winter network, whereas the mean node degree displayed an inverse trend (Supplementary Fig. S8). In addition, the values of three node-level topological features, i.e., degree, betweenness centrality and closeness centrality were significantly higher (*P* < 0.01) in winter than in summer networks. These results indicated that the archaeal OTUs were more connected in winter than in summer.

To examine whether the geographic pattern of networks follows that of community compositions, the subnetwork for each sample from the meta-community network was extracted. Ten topological structure features (the above mentioned eight together with node and edge numbers) of each subnetwork were calculated, and used to generate a distance matrix, which was compared to the geographic distance matrix. The results showed that the network structures did not display significant distance-decay patterns (Supplementary Fig. S9), indicating that spatial shifts in benthic archaeal community composition were incongruent with those in co-occurrence patterns.

## Discussion

### Spatial and seasonal heterogeneity in benthic archaeal community structure

We performed extensive analyses of benthic archaeal communities on 58 surface sediment samples that covered two seasons and a high spatial range: from BS to ECS along the eastern Chinese marginal seas. The abundance of sedimentary archaea was ~10^7^ copies g^−1^, in the range of previously reported values from the same area [39]. This was slightly lower than that from the eutrophic Aarhus Bay (Denmark) [49] but higher than that in the oligotrophic South China Sea [50]. These studies thus indicate the impact of nutrients on benthic archaeal abundance. Previous studies have demonstrated a spatial shift in microbial community structure from estuary to the open sea [e.g., 51, 52]. Similarly, it is shown here that benthic archaeal assemblages differed significantly between CE and adjacent basins. Compared to other areas, CE had an archaeal community with a similar abundance and species diversity, but lower species richness and inter-taxa phylogenetic distances, especially in summer (Fig. 1). These results suggest that the estuarine environmental conditions have selected for several specialized archaeal lineages that are phylogenetically close to each other but have high levels of micro-diversity; the eutrophic nature of the estuary helps to maintain this unique community at a high abundance level. Dominant among these specialized clades in CE were *Bathyarchaeota*, members of which are widespread in anoxic sediments, including estuarine sediments [53], and constitute a total of 25 subgroups according to the 16S rRNA gene phylogeny [54]. The success of *Bathyarchaeota* in estuarine sediments was recently attributed to the presence of lignin, the addition of which was able to stimulate growth [55]. Lignin is a major component of vascular plant cell walls and can accumulate to high concentrations in estuarine sediments due to river runoff [56]. Thus, lignin is likely to be an important determinant for the enrichment of *Bathyarchaeota* in CE.

By comparison, the adjacent sea areas were mainly dominated by *Thaumarchaeota* MG-I and *Woesearchaeota*, which was in line with previous observations [39]. These two clades differed in relative abundance between different sea areas despite comparable total archaeal abundance and diversity. The relative dominance of *Woesearchaeota* in BS and NYS may reflect discharge from the Yellow River. Runoff from this river may have brought a high number of *Woesearchaeota* cells*/*species to the sea as *Woesearchaeota* is widespread in both terrestrial and marine environments and has been reported to be abundant in both the Yellow River water and sediment [57]. This observation supports our previous hypothesis that sediment source is a key driver of the spatial shift in microbial community composition along the Chinese marginal seas [39].

A clear seasonal pattern in archaeal community structure was observed. For example, archaeal taxa in SYS sediments were more abundant in winter than in summer. This was reflected mainly by an increase in the abundance of Group C3 (also referred to the 15th subclade of *Bathyarchaeota*) from summer to winter. Group C3 has been found to increase in abundance [52] and activity [58] in shallow subsurface sediments, supporting the hypothesis that it prefers low-energy environments by H_2_-dependent homoacetogenesis [58, 59]. Generally, the SYS samples would have experienced lower sedimentation rates compared to adjacent areas [38] largely due to the weak impact of river discharges especially in winter. This may have created a relatively low-nutrient environment favoring the growth of Group C3. However, the overall extent of seasonal variation in the benthic archaeal community composition was smaller than that of spatial variation.

### Strong geographic pattern with varying contribution from different lineages

The NMDS analysis confirmed the spatial variation of archaeal assemblages by revealing a clear community separation according to sampling area rather than by season (Fig. 3c). This was consistent with previous reports which found benthic bacterial [60] and microbial eukaryote [61] communities were more variable with sediment depth or region than with seasonal change. In fact, no significant seasonal differences in archaeal community composition were observed in any of the sampling areas studied. This was unexpected as environmental factors, especially temperature, varied greatly between seasons (e.g., the bottom water temperature decreased by ~20 °C from summer to winter in BS; Supplementary Fig. S10). In contrast, in planktonic microbial communities seasonal environmental changes have been found to have a greater effect than biogeography [62, 63]. Such a disparity in the pattern of microbial community dynamics between pelagic and benthic niches suggests a greater temporal habitat stability in sediment than in the water column [64]. Alternatively, the sediment-dwelling microbes may have evolved to withstand temporal environmental changes. The high sedimentation rate in some coastal areas may make environmental adaptation of microorganisms impossible. Specifically, in the eastern Chinese marginal seas, the sedimentation rates ranged between 0 and 42.9 mm/year in areas of mud deposition, and most values were <10 mm/year [38]. These rates were considered to be not very high. Thus, the benthic microbes would have sufficient time to sense and adapt to their environments.

The geographic pattern seen in the benthic archaeal community reflected a clear basin-to-basin separation. This is a pattern similar to that observed in both prokaryote [39, 65] and microbial eukaryote [61] communities from the eastern Chinese marginal seas. Furthermore, the present study demonstrates that underpinning this microbial biogeography was a distance-decay pattern, which was observed in both summer and winter (Fig. 3d). The distance-decay pattern has frequently been found to underly marine microbial spatial dynamics [66]. However, benthic bacteria may display higher decay rates with increasing spatial distance compared to planktonic bacteria [66]. This suggests weaker dispersal abilities for benthic relative to planktonic microorganisms.

Despite this additional information, the contribution of a single microbial group to the whole-community distance-decay pattern is still poorly understood. In this study, it was found that different archaeal taxa exhibited varying distance-decay curves with more abundant clades displaying steeper curves, i.e., stronger distance-decay relationships (Fig. 4). MG-I was the most abundant clade across all samples (Supplementary Fig. S3) and showed the highest rate of community turnover with spatial distance. This rate was followed by those of *Woesearchaeota* and *Bathyarchaeota* that ranked second and third in relative abundance. Thus, MG-I accounted for the highest proportion of the overall distance-decay pattern. However, this appeared to be in contradiction to the observed positive correlation between abundance and occurrence of OTUs (Fig. 3a). It is likely that highly abundant taxa have more chances to disperse nearby [67] resulting in a weak distance-decay pattern. To resolve this discrepancy, the Bray-Curtis dissimilarities for each archaeal clade were compared. MG-I showed the lowest cross-sample community dissimilarities (Fig. 4f) although exhibiting the steepest distance-decay curve. Indeed, this low community dissimilarity may be attributed to an even pattern of site occupancy among MG-I OTUs (Fig. 3b). Inversely, the low site occupancy of most *Woesearchaeota* and *Bathyarchaeota* OTUs may result in their high community dissimilarities. Taken together, these findings suggest that the OTU occurrence pattern is an important driver of community dissimilarity but the extent of community dissimilarity does not necessarily relate to that of a distance-decay relationship.

### Greater role of stochasticity relative to determinism in benthic archaeal community assembly

It is proposed that sedimentary microorganisms may have low dispersal abilities, which may enhance the effect of stochasticity on community assembly. Consistently, using a null model, a stronger role of stochasticity relative to determinism in governing the archaeal community assembly in eastern Chinese marginal sea sediments was detected (Fig. 5b). Furthermore, VPA (Fig. 6) and the partial Mantel test (Supplementary Table S3) corroborated this observation in summer by showing a higher effect of spatial than environmental factors. For the winter samples, the partial Mantel test revealed that spatial factors were overwhelming, which was consistent with the null model. However, this was in contrast to the VPA that showed a slightly higher effect of environmental factors. It should be emphasized that VPA has previously been found to fail to correctly predict the environmental and spatial components of community variations [68–70]. A large proportion of the community variation was unexplained in the VPA, the explanation for which may come from the importance of stochastic processes of growth, death, colonization and extinction [14]. This large unexplained community variation and high autocorrelation between spatial and environmental factors in winter samples of this study (Fig. 6) may well explain the disparity between VPA and other methods. Indeed, these results imply that spatial factors (stochasticity) have a more important role in structuring the benthic archaeal community in summer than in winter.

It has been considered that geographic scales and environmental gradients largely explain the balance between deterministic and stochastic processes [14]. At relatively large spatial scales, deterministic (environmental) factors have been found to exert a greater effect than stochastic (spatial) factors on benthic microeukaryotic communities in marine sandy beaches (up to 12,000 km) [71]. In contrast, Chen et al. [72] reported that microeukaryotic communities in intertidal sediments (a maximum of ~20 km) were more strongly governed by spatial factors. In the present study, the samples covered a spatial range of up to ~1500 km, and the greater role of stochasticity indicated small environmental gradients across this spatial scale and/or weak impacts of environmental changes. This was in contrast to the previous observation of a stronger effect of determinism relative to stochasticity in structuring soil bacteria across 1092 km, which was mainly driven by a large pH gradient [73]. In the coastal sediments targeted here, physical disturbances (e.g., tidal flow, groundwater discharge and mud mobility) occur frequently. Thus, microbial inhabitants may have evolved to endure such environmental dynamics conferring a weak response to deterministic factors. In contrast, in low-energy deep-sea sediments with relatively uniform environments [74], microorganisms may be more sensitive to physiochemical changes, allowing determinism to dominate the community assembly [75]. Therefore, the resistance of microorganisms to environmental changes together with spatial scales and environmental gradients jointly determine the balance of determinism and stochasticity. Also, it is likely that biotic interactions, including virus lysis and predation [76], and unmeasured environmental factors may contribute to the community variation (Fig. 6a, b).

The null model allowed the impact of specific ecological processes within determinism and stochasticity to be discerned. In fact, it was found that the assembly of benthic archaeal community was mainly controlled by dispersal limitation, homogeneous selection and drift and this was the situation in both winter and summer (Fig. 5a). Dispersal limitation accounted for ~40% of the community variation across all samples. This proportion was higher than that reported for lake bacteria [26, 77] and surface-ocean prokaryotes [78]. However, this is considered reasonable because sediment has a lower regional connectivity than water, which decreases the probability of active dispersal [66]. In our study sites the existence of several sedimentary patches that result from river discharges could elevate the contribution of dispersal limitation. Homogeneous selection is one process in which environments constrain the divergence of microbial populations [28]. The prevalence of this process has also been found in lake bacterial (>70%) [26, 77] and surface-ocean prokaryotic (~23–24% of the community variation) [78] communities. The indication is that several consistent environmental factors (e.g., the concentration (C/N) and source (δ^13^C) of organic matter that varied slightly across samples and/or unmeasured factors in this study) selected for similar microbial communities across samples. The enhanced abilities of benthic archaea for adapting to environmental dynamics act as another possible explanation. Recently, Wu et al. [79] reported that dispersal limitation accounted for 33.3% of the protist community turnover in deep-sea sediments of the South China Sea whereas homogeneous dispersal explained 0%. The relative contribution of these two processes was similar to that reported here. Also, Wu et al. [79] showed that drift provided an explanatory power of 13.3%, a proportion lower than observed here. Drift occurs in communities with relatively low population sizes and inhabiting relatively stable environments [80]. The archaeal populations studied here represent a low-size community compared to bacteria [39] but the environmental conditions in coastal sediments could be dynamic. These factors may jointly determine the relative contribution of drift.

Delineating OTUs at different similarity levels affected the quantification of ecological processes (Fig. 5, Supplementary Fig. S7), although a consistent dominance of stochasticity relative to determinism was observed. Drift provided a lower explanatory power in the analysis based on the 99% cut-off than that based on the 97% cut-off, whereas an opposite trend was seen for dispersal limitation. OTUs clustered at different cut-offs may represent different taxonomic units with varying evolutionary rates, and thus may assemble under different mechanisms [78]. Additionally, the fluctuations may reflect the sensitivity of the null model analysis to the OTU number, in particular for the low-size archaeal population. The higher number of OTUs (species) obtained at the 99% cut-off compared to the 97% cut-off may represent a relatively higher population size, under which condition drift is less likely to occur. In turn, defining OTUs at a high similarity level leads to a decreased sequence number (occurrence frequency) for each OTU, which may lower the possibility of dispersal.

### Co-occurrence network: seasonality and unmatched spatial dynamics with community composition

Complex interactions occur within a microbial community which may be partially revealed by co-occurrence networks [12, 32]. The spatiotemporal variations of network-level topological features were investigated here to explore whether shifts in archaeal community compositions could determine their co-occurrence patterns. The results revealed a clear seasonal pattern of associations; the archaeal taxa became more connected with one another from summer to winter. One explanation may be attributed to a lower summer spatial connectivity, which was mainly contributed by the strong river discharges (resulting in different sedimentary patches) and the occurrence of the Yellow Sea Cold Water Mass (appear in summer and disappear in winter) [81]. These events may make different sampling areas geographically more separated thus resulting in an overall more scattered co-occurrence pattern in summer. Indeed, this was consistent with the observation of a higher effect of spatial factors in summer than in winter (Fig. 6a, b). The disparity in sample number between seasons may add some uncertainty to the data.

Conversely, although there was a clear shift in archaeal community composition along with geographic distance, no congruent spatial change was seen in the co-occurrence patterns (Supplementary Fig. S9). These results provide evidence that the dynamics of microbial community compositions and co-occurrence patterns are asynchronous and are not necessarily correlated. This will lead to a better understanding of microbial ecology. Caution is needed insofar as the estimated variations in co-occurrence patterns that were derived from a topology-based system approach did not reflect true inter taxa correlations [33]. Thus, further efforts are needed to identify specific taxa–taxa interactions and link them to community compositions and further ecological functions.

## Conclusion

This study demonstrated clear spatiotemporal patterns in benthic archaeal community composition, geographic distribution, underlying mechanism and co-occurrence relationship using surface sediment samples collected from the eastern Chinese marginal seas. The results showed a significant seasonal-consistent distance-decay pattern in the benthic archaeal community, which was found in different lineages with their respective contribution positively related to relative abundance. Furthermore, we provided evidence that stochasticity had a greater role than determinism in structuring this distance-decay pattern. More importantly, a quantitative assessment of ecological processes revealed that dispersal limitation, homogeneous selection and drift exerted important roles in the community assembly of sedimentary archaea; such a scenario experienced little impact from seasonal change. This may be due to the distinction in sediment source of each sampling area, similarity in environmental conditions across areas, and/or high resistance of benthic archaea to environmental disturbances. It is also shown that the archaeal co-occurrence relationships changed over seasons. Moreover, their turnover with spatial distance was incongruent with that of community compositions providing important implications for the dynamics of archaeal communities and inter taxa interactions in coastal sediments.

## Supplementary information

Supplementary information
