# Supplementary material for: Spatiotemporal dynamics of the archaeal community in coastal sediments: assembly process and co-occurrence relationship
Source: ISME J. 2020 Mar 4;14(6):1463–78. doi: 10.1038/s41396-020-0621-7 (PMC7242467; doi:10.1038/s41396-020-0621-7)
Supplement: Supplementary file 1 — Supplementary information [file 41396_2020_621_MOESM1_ESM.docx]

**Supplementary information**

**Spatiotemporal dynamics of the archaeal community in coastal sediments: assembly process and co-occurrence relationship**

Jiwen Liu^1,2,3^, Shangqing Zhu^1^, Xiaoyue Liu^1^, Peng Yao^2,4^, Tiantian Ge^5^, Xiao-Hua Zhang^1,2,3*^

Corresponding author: Xiao-Hua Zhang; Email: xhzhang@ouc.edu.cn

**This file includes:**

Figures S1 to S10

Tables S1 to S4

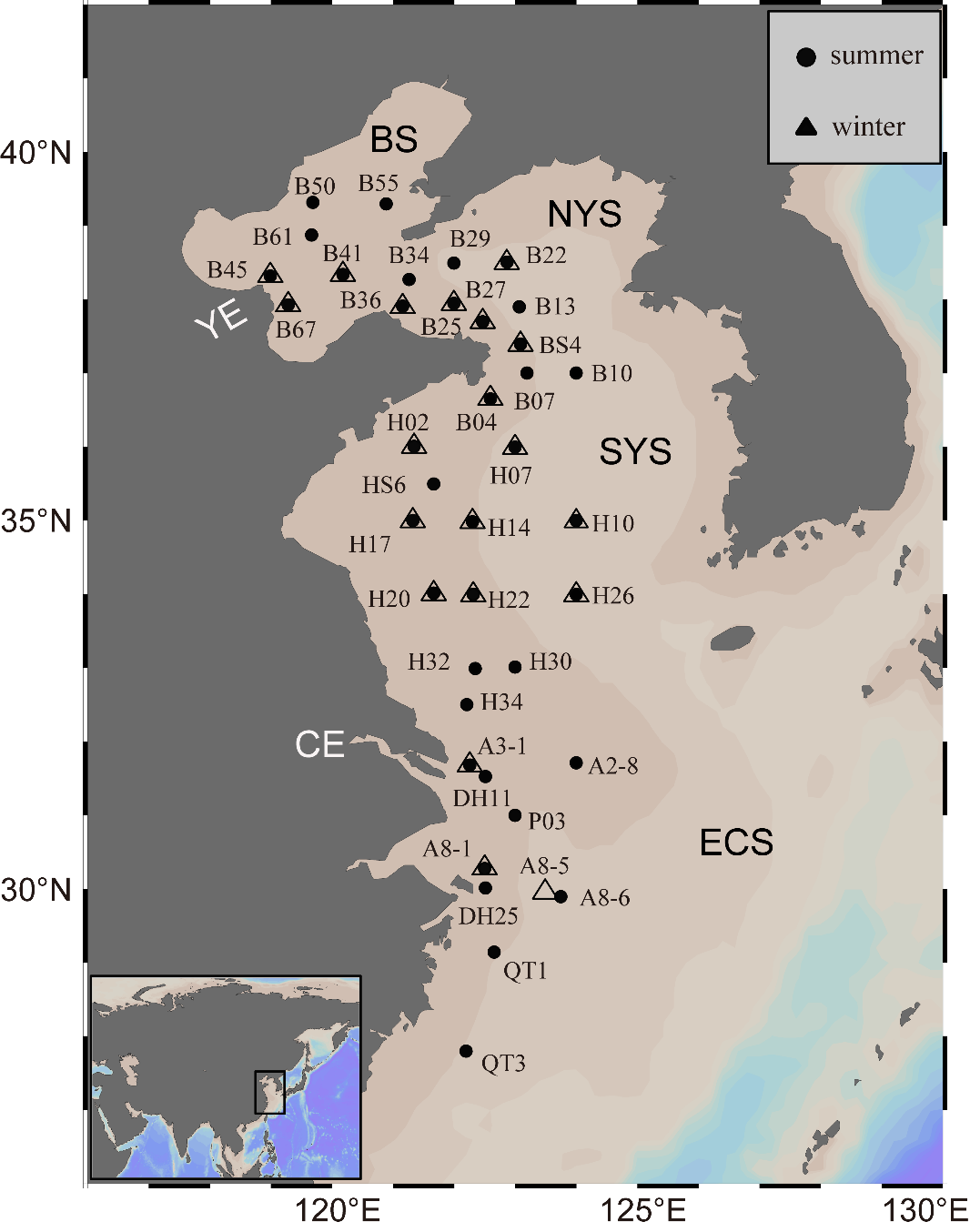


**Supplementary Fig. S1** Sampling map in the eastern Chinese marginal seas. Sites marked by both circle and triangle indicate that both the summer and winter samples were available. BS, Bohai Sea; NYS, north Yellow Sea; SYS, south Yellow Sea; ECS, East China Sea; YE, Yellow Estuary; CE, Changjiang Estuary.


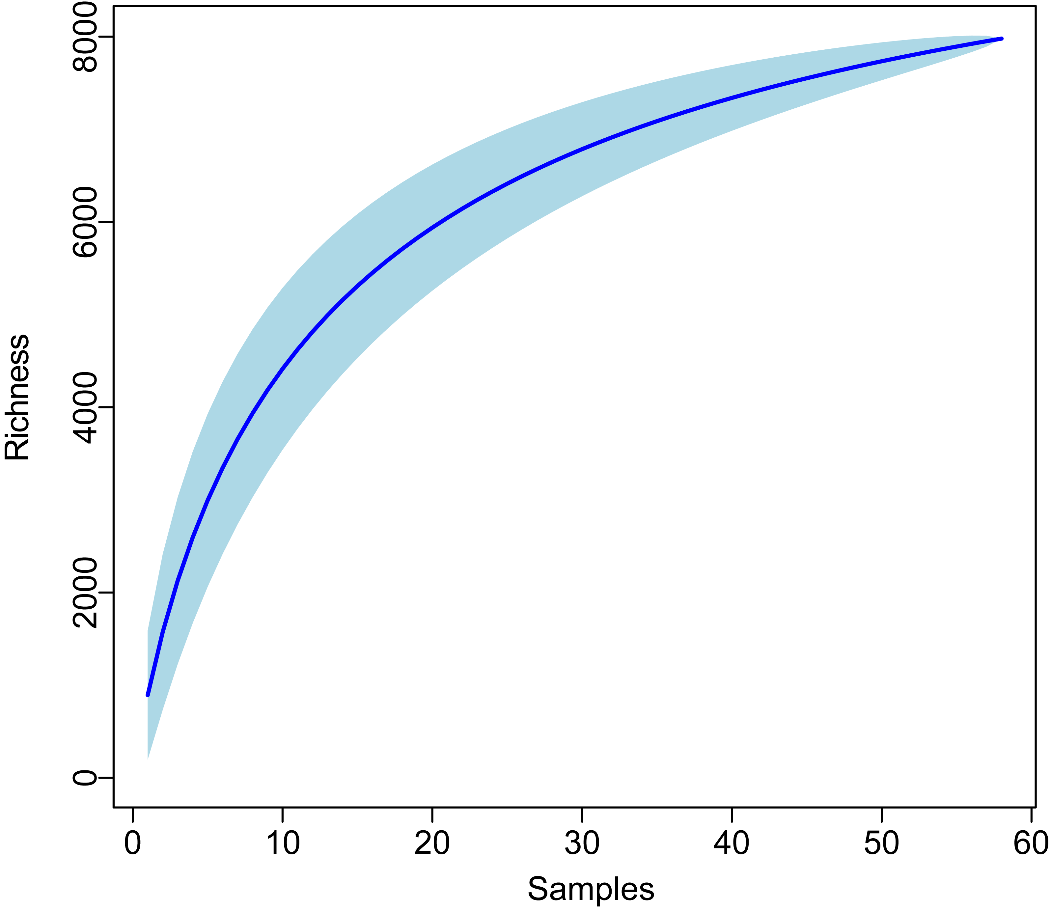


**Supplementary Fig. S2** Species accumulation curve based on the OTU level community.


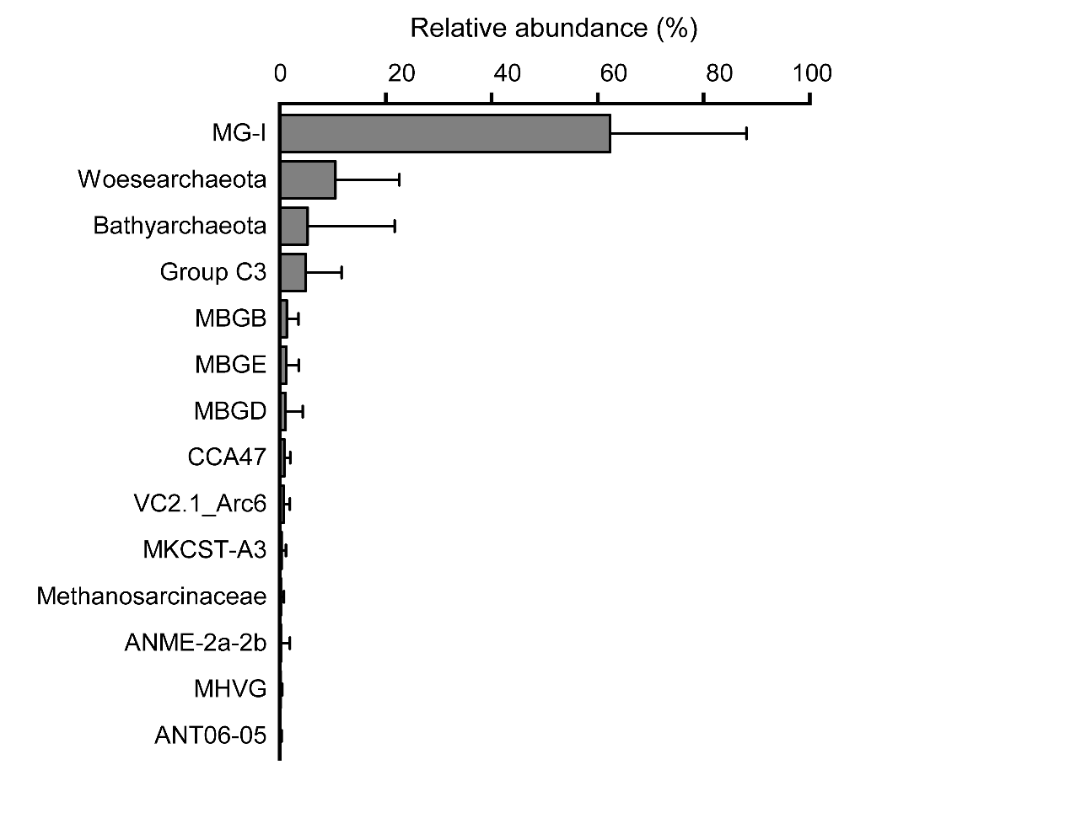


**Supplementary Fig. S3** Mean relative abundance of the most abundant archaeal clades across all samples.


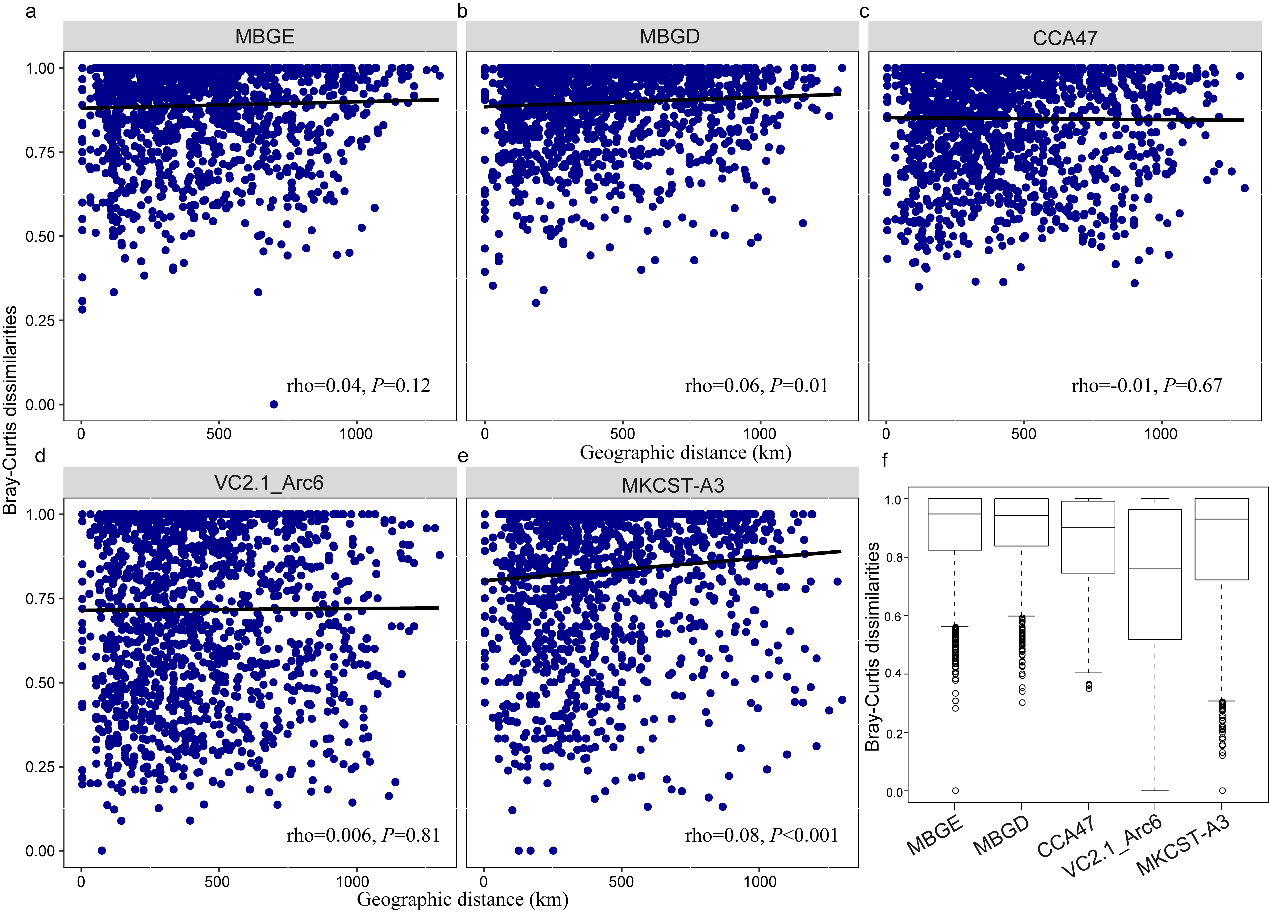


**Supplementary Fig. S4** Distance-decay patterns of the top sixth to tenth most abundant archaeal clades (rank in relative abundance, **a-e**) and their respective OTU-level Bray-Curtis dissimilarities (**f**).


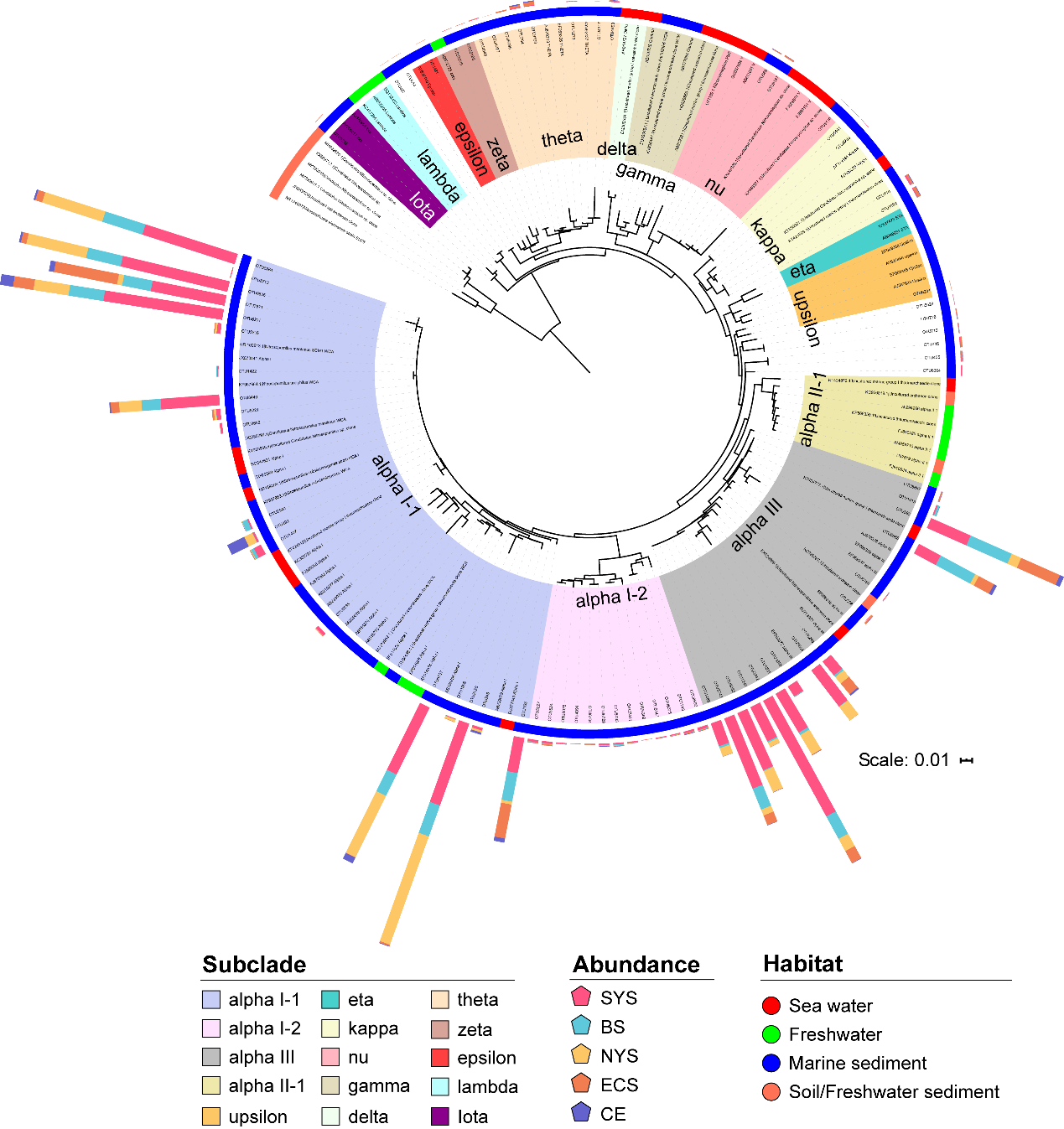


**Supplementary Fig. S5** Neighbor-joining phylogenetic tree showing the clustering relationships of MG-I OTUs. The outer color circle around the phylogenetic tree suggests different habitats. The bar plot suggests the accumulative abundance of each OTU across sampling areas. The subclades of MG-I were designated following the nomenclature reported previously [1, 2]. A new cluster i.e., alpha I-2 was identified; this cluster was exclusively comprised of OTUs retrieved in this study.


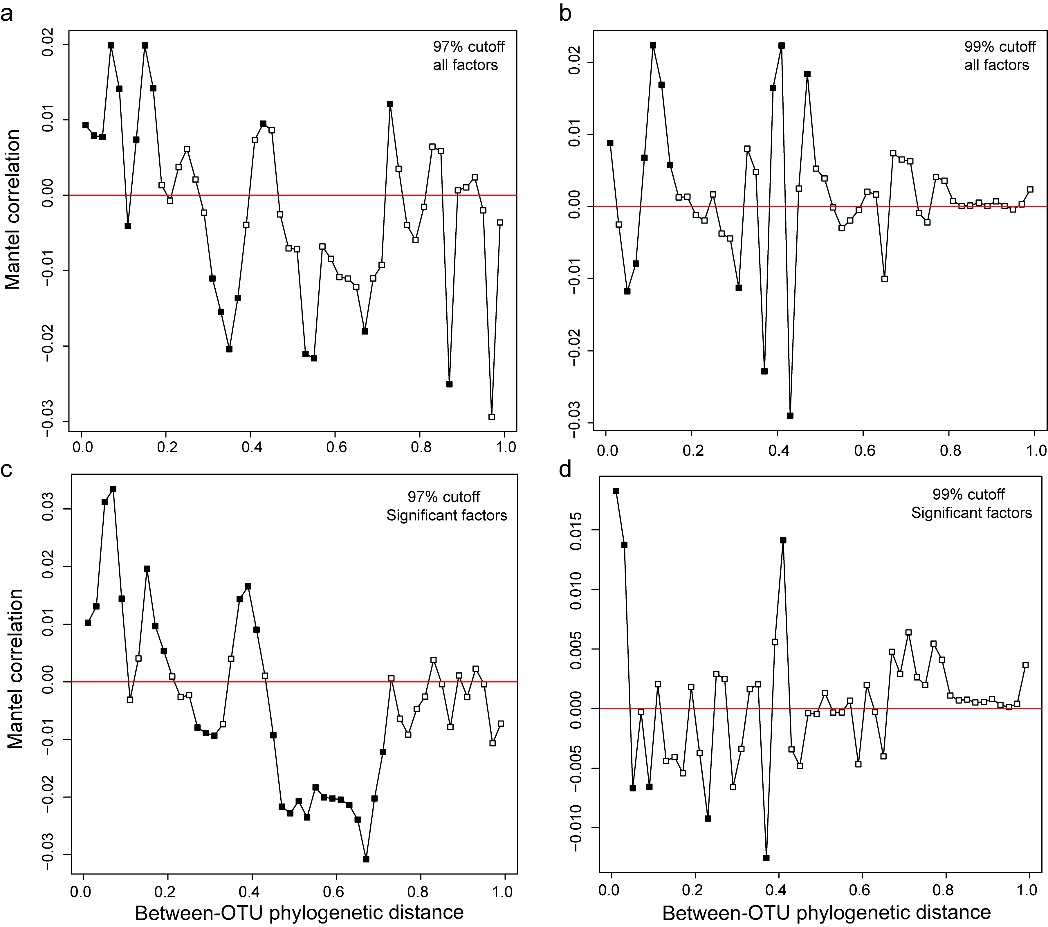


**Supplementary Fig. S6** Phylogenetic Mantel correlogram evaluating phylogenetic signal in the benthic archaeal communities. **a (b)** OTUs at a 97% (99%) similarity level and environmental optima based on all environmental factors; **c (d)** OTUs at a 97% (99%) similarity level and environmental optima based on only significant environmental factors. Solid symbols indicate significant phylogenetic signals (*P* < 0.05, 999 permutations). The phylogenetic distances were partitioned into 50 classes by 0.02 units.


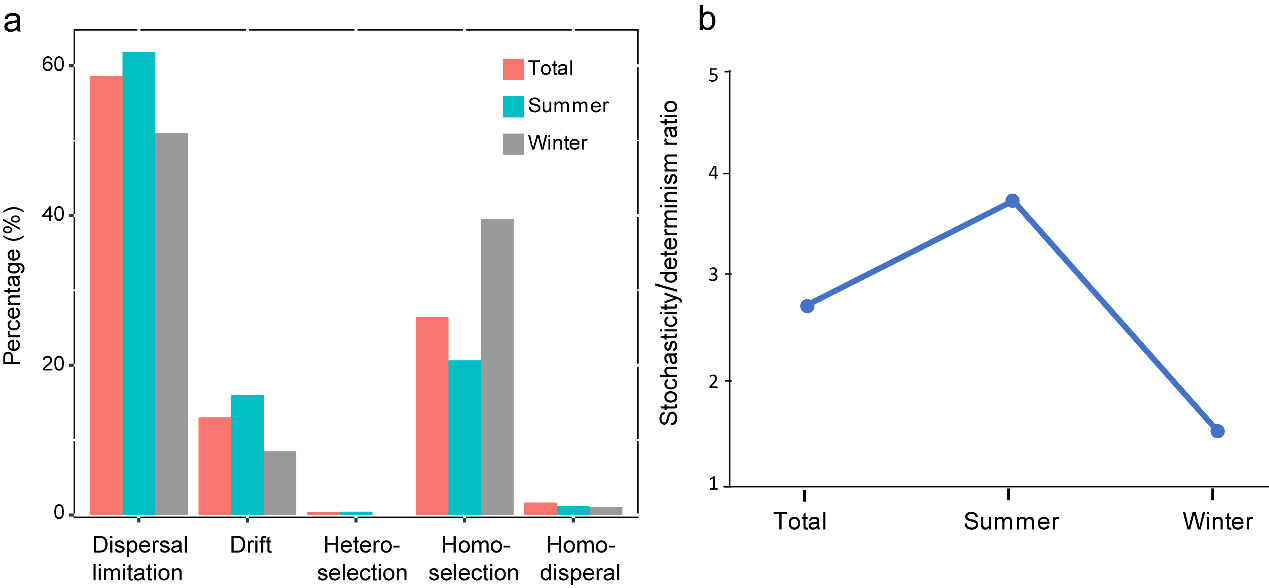


**Supplementary Fig. S7** Null model analysis based on OTUs defined at a 99% similarity level revealing the relative contribution of different ecological processes (**a**) and the ratio of stochasticity and determinism (**b**) in governing the assembly of archaeal communities in summer, winter and across all samples.


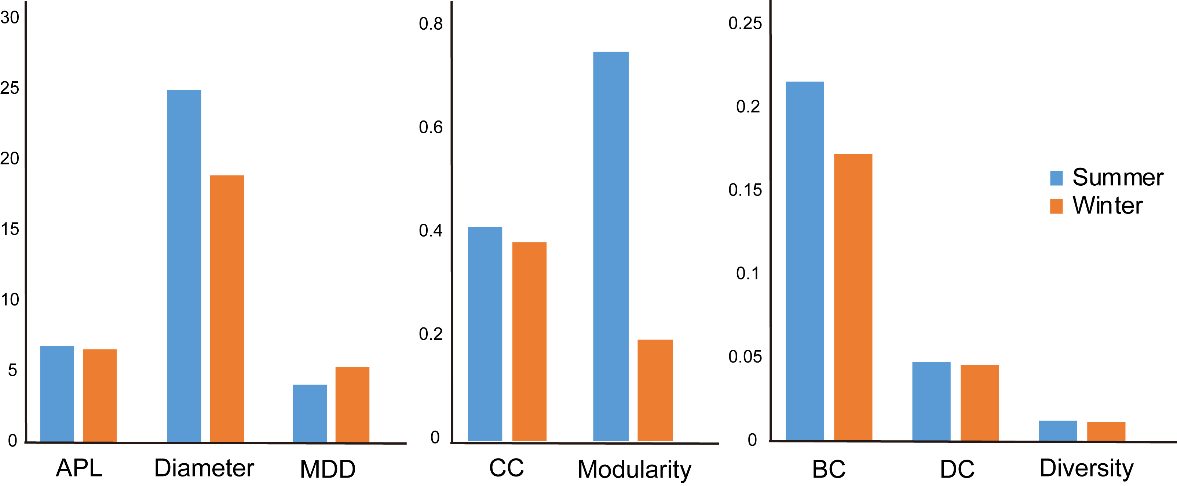


**Supplementary Fig. S8** Network-level topological features of networks in winter and summer. APL, average path length; MDD, mean node degree; CC, clustering coefficient; BC, betweenness centralization; DC, degree centralization.


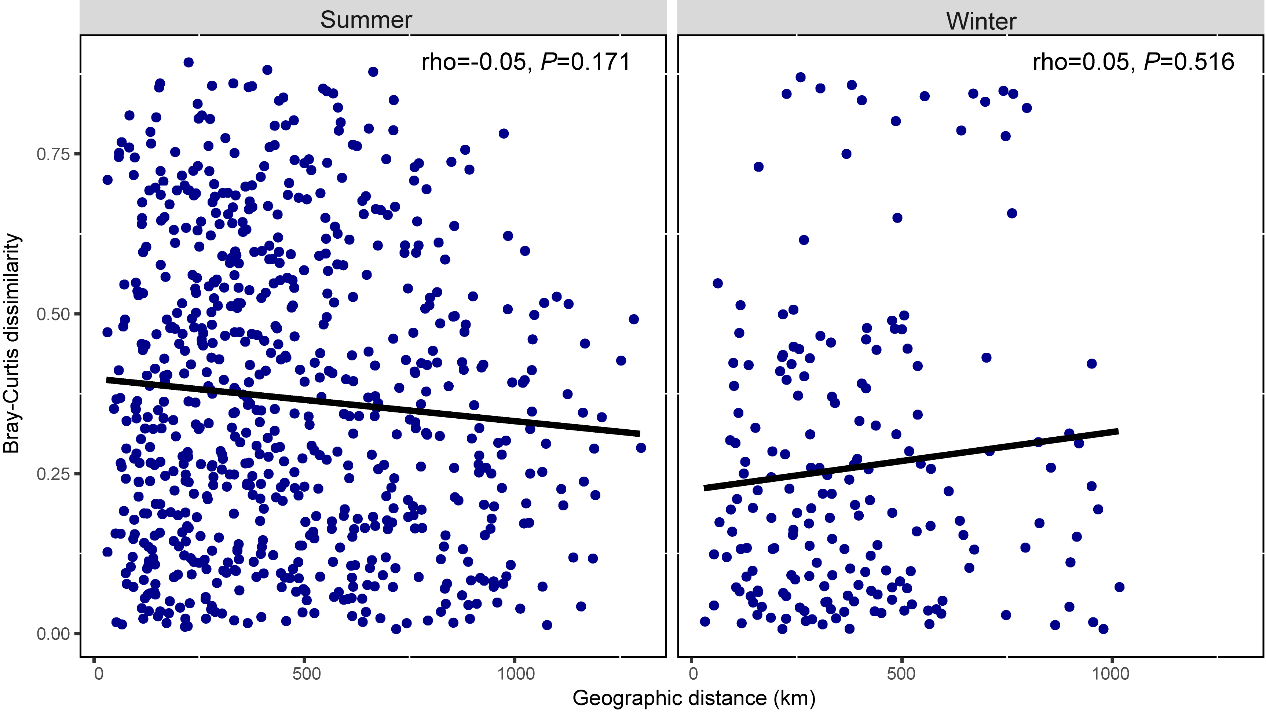


**Supplementary Fig. S9** Distance-decay patterns of co-occurrence relationships represented by ten network-level topological structure features.


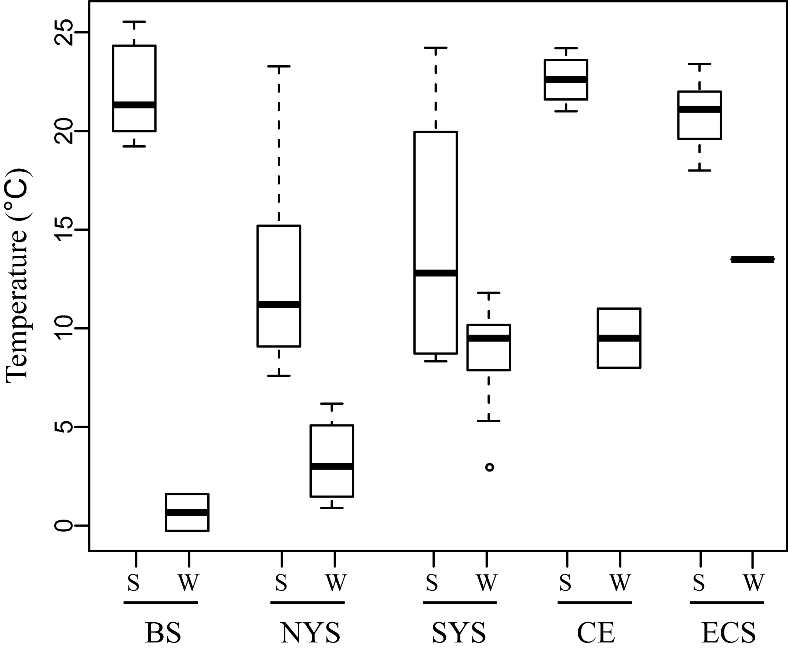


**Supplementary Fig. S10** Seasonal variation of bottom water temperature in the studied areas. S, summer; W, winter.

**Supplementary Table S1** The number of rarefied reads, OTUs, Good’s coverage, richness (Chao I and Ace) and diversity (Shannon and Simpson) indices across all samples at the 97% similarity level

| Area | Station | Read number | | OTUs | Ace | Chao I | Coverage | Shannon | Simpson |
| --- | --- | --- | --- | --- | --- | --- | --- | --- | --- |
| BS | B41W | | 12630 | 1077 | 2817 | 2193 | 0.95 | 4.11 | 0.05 |
|  | B41S | | 12630 | 589 | 1432 | 981 | 0.98 | 3.04 | 0.13 |
|  | B45W | | 12630 | 850 | 1943 | 1472 | 0.97 | 3.74 | 0.07 |
|  | B45S | | 12630 | 655 | 1180 | 1185 | 0.97 | 3.27 | 0.09 |
|  | B50S | | 12630 | 830 | 1721 | 1397 | 0.97 | 4.33 | 0.03 |
|  | B55S | | 12630 | 1112 | 2084 | 1773 | 0.96 | 4.89 | 0.03 |
|  | B61S | | 12630 | 1443 | 3375 | 2574 | 0.94 | 4.88 | 0.03 |
|  | B67W | | 12630 | 998 | 2378 | 1751 | 0.96 | 4.48 | 0.04 |
|  | B67S | | 12630 | 471 | 1437 | 935 | 0.98 | 2.48 | 0.18 |
| NYS | B13S | | 12630 | 455 | 976 | 783 | 0.98 | 3.10 | 0.12 |
|  | B22W | | 12630 | 720 | 1572 | 1151 | 0.97 | 3.26 | 0.09 |
|  | B22S | | 12630 | 739 | 1627 | 1265 | 0.97 | 3.68 | 0.06 |
|  | B25W | | 12630 | 1189 | 1837 | 1790 | 0.96 | 4.42 | 0.05 |
|  | B25S | | 12630 | 1257 | 2455 | 2044 | 0.95 | 4.59 | 0.04 |
|  | B27W | | 12630 | 1076 | 1583 | 1521 | 0.97 | 4.62 | 0.04 |
|  | B27S | | 12630 | 836 | 1574 | 1262 | 0.97 | 3.86 | 0.07 |
|  | B29S | | 12630 | 943 | 2016 | 1591 | 0.96 | 3.22 | 0.23 |
|  | B34S | | 12630 | 984 | 2046 | 1650 | 0.96 | 3.50 | 0.13 |
|  | B36W | | 12630 | 1058 | 1579 | 1522 | 0.97 | 4.49 | 0.04 |
|  | B36S | | 12630 | 810 | 2497 | 1491 | 0.96 | 3.48 | 0.09 |
| SYS | H02W | | 12630 | 1310 | 3360 | 2270 | 0.94 | 4.17 | 0.07 |
|  | H02S | | 12630 | 738 | 1913 | 1444 | 0.97 | 2.84 | 0.20 |
|  | H07W | | 12630 | 906 | 2825 | 1830 | 0.96 | 3.82 | 0.08 |
|  | H07S | | 12630 | 773 | 2029 | 1414 | 0.97 | 3.54 | 0.06 |
|  | H10W | | 12630 | 835 | 1642 | 1418 | 0.97 | 4.49 | 0.04 |
|  | H10S | | 12630 | 297 | 1053 | 640 | 0.99 | 3.16 | 0.08 |
|  | H14W | | 12630 | 815 | 2118 | 1462 | 0.96 | 3.18 | 0.15 |
|  | H14S | | 12630 | 1039 | 2188 | 1777 | 0.96 | 4.29 | 0.05 |
|  | H17W | | 12630 | 1425 | 2978 | 2329 | 0.95 | 5.62 | 0.01 |
|  | H17S | | 12630 | 1610 | 2210 | 2266 | 0.95 | 5.82 | 0.01 |
|  | H20W | | 12630 | 466 | 1488 | 968 | 0.98 | 2.51 | 0.19 |
|  | H20S | | 12630 | 688 | 2217 | 1447 | 0.97 | 3.18 | 0.10 |
|  | H22W | | 12630 | 1267 | 2407 | 1980 | 0.96 | 4.96 | 0.03 |
|  | H22S | | 12630 | 921 | 2972 | 1904 | 0.96 | 3.49 | 0.08 |
|  | H26W | | 12630 | 1154 | 3409 | 2256 | 0.95 | 4.83 | 0.03 |
|  | H26S | | 12630 | 1295 | 3023 | 2211 | 0.95 | 4.45 | 0.04 |
|  | H30S | | 12630 | 856 | 2463 | 1633 | 0.96 | 3.55 | 0.07 |
|  | H32S | | 12630 | 1043 | 2583 | 1880 | 0.96 | 3.96 | 0.08 |
|  | H34S | | 12630 | 446 | 1716 | 1102 | 0.98 | 2.64 | 0.15 |
|  | HS6S | | 12630 | 366 | 2088 | 967 | 0.98 | 2.53 | 0.15 |
|  | BS4W | | 12630 | 941 | 2362 | 1605 | 0.96 | 3.53 | 0.10 |
|  | BS4S | | 12630 | 1506 | 3171 | 2551 | 0.94 | 5.15 | 0.03 |
|  | B04W | | 12630 | 1779 | 3442 | 2797 | 0.94 | 5.69 | 0.02 |
|  | B04S | | 12630 | 1026 | 3068 | 2106 | 0.96 | 4.34 | 0.04 |
|  | B07S | | 12630 | 485 | 1088 | 808 | 0.98 | 3.04 | 0.11 |
|  | B10S | | 12630 | 608 | 1311 | 1003 | 0.98 | 3.29 | 0.12 |
| CE | A3_1W | | 12630 | 182 | 504 | 326 | 0.99 | 2.59 | 0.16 |
|  | A3_1S | | 12630 | 330 | 1050 | 631 | 0.99 | 2.66 | 0.19 |
|  | A8_1W | | 12630 | 604 | 1541 | 1023 | 0.98 | 3.50 | 0.11 |
|  | A8_1S | | 12630 | 701 | 1230 | 1019 | 0.98 | 4.55 | 0.04 |
|  | DH11S | | 12630 | 956 | 2036 | 1600 | 0.96 | 4.93 | 0.02 |
|  | DH25S | | 12630 | 352 | 441 | 439 | 0.99 | 4.18 | 0.03 |
| ECS | A2_8S | | 12630 | 890 | 2371 | 1558 | 0.96 | 3.84 | 0.10 |
|  | A8_5W | | 12630 | 1066 | 2464 | 1798 | 0.96 | 4.49 | 0.04 |
|  | A8_6S | | 12630 | 947 | 1951 | 1537 | 0.96 | 3.75 | 0.12 |
|  | P03S | | 12630 | 1274 | 3455 | 2373 | 0.94 | 4.02 | 0.11 |
|  | QT1S | | 12630 | 1367 | 3321 | 2392 | 0.95 | 5.13 | 0.02 |
|  | QT3S | | 12630 | 467 | 1982 | 1080 | 0.98 | 3.17 | 0.08 |

**Supplementary** **Table S2** Community comparison based on the PERMANOVA analysis

|  | BS+NYS | SYS | CE | ECS |
| --- | --- | --- | --- | --- |
| BS+NYS |  |  |  |  |
| SYS | R^2^=0.052  *P*=0.024 |  |  |  |
| CE | R^2^=0.202  *P*=0.006 | R^2^=0.147  *P*=0.006 |  |  |
| ECS | R^2^=0.199  *P*= 0.006 | R^2^=0.118  *P*=0.006 | R^2^=0.265 *P*=0.018 |  |

**Supplementary** **Table S3** Partial Mantel test between spatial and environmental factors (r, Mantel statistics; *P*, significance). Geographic distance was calculated from longitude and latitude in R

|  | Test | Statistics |
| --- | --- | --- |
| Summer | Spatial factor, environmental factor controlled | *r* = 0.308, *P* = 0.001 |
|  | Environmental factor, spatial factor controlled | *r* = 0.295, *P* = 0.001 |
| Winter | Spatial factor, environmental factor controlled | *r* = 0.266, *P* = 0.001 |
|  | Environmental factor, spatial factor controlled | *r* = 0.001, *P* = 0.396 |

**Supplementary Table S4** Environmental characterization of bottom water (temperature, salinity, DO), porewater (PO_4_^3-^, NO_2_^-^, SiO_3_^2-^, NH_4_^+^) and sediment particle

| Area | Station | Water depth  (m) | Temperature (°C) | Salinity | DO  (mg/L) | PO_4_^3-^  (μmol/L) | NO_2_^-^  (μmol/L) | SiO_3_^2-^  (μmol/L) | NH_4_^+^  (μmol/L) | TN% | TOC% | C/N | Sand  (> 63 μm) | Silt  (4-63 μm) | Clay  (< 4 μm) | Median size (μm) | δ^13^C  (‰) |
| --- | --- | --- | --- | --- | --- | --- | --- | --- | --- | --- | --- | --- | --- | --- | --- | --- | --- |
|  | B41W | 27.00 | 1.60 | 32.26 | 11.27 | 1.17 | 0.61 | 48.68 | 165.75 | 0.03 | 0.26 | 9.26 | 55.55 | 34.72 | 9.74 | 72.43 | -22.27 |
|  | B41S | 27.00 | 20.00 | 31.17 | 3.94 | 1.90 | 0.11 | 100.15 | 9.22 | 0.06 | 0.37 | 7.65 | 43.35 | 43.40 | 13.25 | 52.87 | -22.26 |
|  | B45W | 20.00 | -0.269 | NA | 12.05 | 0.53 | 0.41 | 32.49 | 15.33 | 0.07 | 0.47 | 8.00 | 5.47 | 73.57 | 20.96 | 14.26 | -22.29 |
|  | B45S | 20.50 | 24.32 | 31.01 | 5.44 | 0.75 | 0.17 | 63.41 | 19.40 | 0.08 | 0.46 | 7.10 | 9.69 | 68.90 | 21.41 | 13.69 | -22.48 |
| BS | B50S | 25.50 | 20.09 | 31.16 | 2.15 | 2.40 | 0.13 | 114.92 | 16.22 | 0.12 | 0.77 | 7.79 | 16.79 | 55.38 | 27.84 | 9.34 | -22.95 |
|  | B55S | 32.80 | 19.22 | 31.29 | 6.29 | 2.02 | 0.54 | 64.07 | 9.61 | 0.04 | 0.32 | 8.83 | 56.26 | 31.12 | 12.62 | 90.20 | -22.56 |
|  | B61S | 26.00 | 22.56 | 31.02 | 3.96 | 8.73 | 0.27 | 191.99 | 27.81 | 0.13 | 0.94 | 8.44 | 9.77 | 64.95 | 25.28 | 11.04 | -22.32 |
|  | B67W | 12.00 | NA | NA | NA | 0.17 | 0.35 | 49.21 | 127.46 | 0.06 | 0.44 | 8.49 | 2.32 | 75.19 | 22.50 | 12.95 | -23.26 |
|  | B67S | 12.00 | 23.50 | 30.40 | 6.40 | 0.70 | 0.12 | 108.30 | 27.90 | 0.07 | 0.45 | 7.79 | 9.43 | 71.73 | 18.84 | 14.53 | -23.18 |
|  | B13S | 61.00 | 7.59 | 32.13 | 7.38 | 1.66 | 0.21 | 82.37 | 4.50 | 0.05 | 0.35 | 8.80 | 53.48 | 36.16 | 10.36 | 68.83 | -22.64 |
|  | B22W | 55.00 | 6.18 | 32.34 | 9.98 | 1.59 | 0.42 | 100.74 | 70.85 | 0.08 | 0.52 | 7.97 | 30.34 | 56.43 | 13.23 | 42.35 | -22.27 |
|  | B22S | 56.50 | 9.64 | 32.17 | 7.73 | 1.62 | 0.20 | 137.34 | 4.87 | 0.10 | 0.64 | 7.37 | 30.36 | 55.30 | 14.35 | 40.30 | -22.41 |
|  | B25W | 28.00 | 2.04 | 32.25 | 11.15 | 0.93 | 0.28 | 92.90 | 172.29 | 0.06 | 0.41 | 7.63 | 16.67 | 67.91 | 15.42 | 30.42 | -21.95 |
|  | B25S | 27.00 | 18.80 | 31.40 | 4.50 | 1.90 | 0.12 | 106.50 | 17.40 | 0.08 | 0.51 | 7.10 | 18.37 | 66.19 | 15.40 | 27.59 | -22.13 |
| NYS | B27W | 42.50 | 3.98 | 32.20 | 10.59 | 1.19 | 0.08 | 93.32 | 43.08 | 0.16 | 1.17 | 8.28 | 8.18 | 70.86 | 20.96 | 17.23 | -22.07 |
|  | B27S | 43.00 | 11.60 | 31.80 | 5.90 | 3.90 | 0.17 | 169.40 | 15.00 | 0.17 | 1.27 | 8.70 | 19.68 | 60.93 | 19.40 | 23.37 | -22.36 |
|  | B29S | 49.50 | 8.52 | 32.18 | 7.93 | 2.26 | 0.20 | 168.74 | 9.13 | 0.10 | 0.66 | 7.59 | 49.75 | 39.54 | 10.71 | 63.48 | -22.37 |
|  | B34S | 40.10 | 11.20 | 31.90 | 7.00 | 3.50 | 0.12 | 149.10 | 22.00 | 0.04 | 0.26 | 6.80 | 69.15 | 22.78 | 8.10 | 104.16 | -22.80 |
|  | B36W | 19.50 | 0.90 | 32.07 | 11.49 | 0.79 | 0.26 | 60.86 | 27.28 | 0.06 | 0.43 | 8.38 | 6.95 | 72.54 | 20.51 | 18.76 | -21.95 |
|  | B36S | 20.50 | 23.28 | 30.78 | 6.41 | 1.19 | 0.11 | 85.33 | 7.75 | 0.05 | 0.36 | 7.98 | 14.87 | 69.70 | 15.43 | 26.17 | -22.33 |
|  | H02W | 38.00 | 8.90 | 31.90 | 10.11 | 1.99 | 1.70 | 80.21 | 25.45 | 0.05 | 0.34 | 8.26 | 38.95 | 45.67 | 15.39 | 51.67 | -21.86 |
|  | H02S | 34.00 | 12.80 | 31.50 | 7.00 | 2.90 | 0.08 | 116.90 | 16.00 | 0.05 | 0.33 | 7.50 | 34.31 | 48.54 | 17.20 | 42.18 | -22.20 |
|  | H07W | 70.00 | 9.51 | 32.22 | 8.73 | 2.01 | 0.09 | 97.09 | 26.48 | 0.17 | 1.14 | 7.76 | 0.46 | 54.42 | 45.13 | 4.54 | -21.88 |
|  | H07S | 71.00 | 8.33 | 32.65 | 7.17 | 3.25 | 0.10 | 141.97 | 5.93 | 0.20 | 1.27 | 7.54 | 6.98 | 50.22 | 42.80 | 4.87 | -22.34 |
|  | H10W | 81.00 | 9.64 | 33.03 | 5.92 | 1.53 | 0.09 | 107.70 | 27.59 | 0.10 | 0.63 | 7.61 | 26.25 | 46.87 | 26.89 | 11.21 | -22.06 |
|  | H10S | 81.50 | 8.70 | 32.96 | 6.84 | 2.98 | 0.31 | 143.98 | 3.61 | 0.09 | 0.58 | 7.52 | 38.47 | 42.79 | 18.74 | 35.07 | -22.34 |
|  | H14W | 61.00 | 10.54 | 32.09 | 8.66 | 1.40 | 0.20 | 83.95 | 29.70 | 0.13 | 0.92 | 8.09 | 2.48 | 64.93 | 32.59 | 7.17 | -22.06 |
|  | H14S | 58.00 | 9.00 | 32.60 | 6.50 | 2.40 | 0.14 | 100.60 | 18.80 | 0.13 | 0.84 | 7.60 | 4.75 | 67.43 | 27.80 | 8.03 | -22.42 |
|  | H17W | 35.50 | 9.50 | 31.83 | 9.36 | 2.18 | 0.25 | 62.25 | 237.21 | 0.02 | 0.19 | 8.82 | 81.48 | 12.21 | 6.31 | 153.89 | -22.77 |
|  | H17S | 36.00 | 15.32 | 31.50 | 6.36 | 4.05 | 0.25 | 115.72 | 11.26 | 0.02 | 0.19 | 9.06 | 81.92 | 12.34 | 5.74 | 176.53 | -23.25 |
|  | H20W | 20.00 | 7.88 | 31.69 | 9.71 | 0.70 | 0.51 | 19.28 | 14.97 | 0.02 | 0.16 | 9.08 | 35.26 | 56.81 | 7.94 | 48.52 | -23.55 |
| SYS | H20S | 20.00 | 24.22 | 30.73 | 7.10 | 0.56 | 0.22 | 56.43 | 64.39 | 0.03 | 0.26 | 9.52 | 42.61 | 50.31 | 7.07 | 54.67 | -24.21 |
|  | H22W | 39.50 | 10.17 | 31.87 | 9.21 | 1.26 | 1.60 | 7.67 | 40.02 | 0.03 | 0.24 | 8.46 | 41.84 | 47.78 | 10.38 | 51.17 | -23.35 |
|  | H22S | 37.00 | 13.20 | 32.20 | 7.20 | 3.30 | 0.40 | 105.10 | 80.00 | 0.03 | 0.26 | 11.50 | 45.15 | 47.52 | 7.30 | 57.13 | -23.54 |
|  | H26W | 78.50 | 11.80 | 32.82 | 8.53 | 1.54 | 0.10 | 77.83 | 15.92 | 0.09 | 0.54 | 7.30 | 17.99 | 52.64 | 29.37 | 7.41 | -21.18 |
|  | H26S | 79.00 | 8.74 | 32.99 | 6.79 | 2.31 | 0.15 | 137.73 | 5.12 | 0.10 | 0.60 | 6.87 | 23.77 | 52.06 | 24.18 | 10.36 | -22.07 |
|  | H30S | 34.50 | 20.84 | 30.68 | 3.25 | 0.97 | 0.15 | 69.49 | 7.34 | 0.06 | 0.41 | 7.39 | 35.44 | 47.54 | 17.02 | 25.73 | -21.27 |
|  | H32S | 23.00 | 20.60 | 30.60 | 3.30 | 2.20 | 0.18 | 130.20 | 26.30 | 0.05 | 0.36 | 8.00 | 21.39 | 64.89 | 13.70 | 28.92 | -21.48 |
|  | H34S | 26.00 | NA | NA | 3.67 | 1.98 | 0.21 | 157.47 | 39.13 | 0.06 | 0.36 | 7.38 | 19.23 | 67.61 | 13.16 | 30.56 | -21.68 |
|  | HS6S | 44.00 | 11.95 | 31.67 | 6.10 | 1.73 | 0.19 | 128.02 | 3.96 | 0.04 | 0.31 | 9.24 | 56.29 | 31.46 | 12.26 | 71.74 | -22.33 |
|  | BS4W | 59.00 | 5.30 | 31.99 | 10.25 | 1.39 | 0.46 | 76.53 | 17.82 | 0.06 | 0.46 | 8.39 | 28.69 | 57.60 | 13.71 | 39.30 | -22.71 |
|  | BS4S | 54.50 | 23.16 | 32.02 | 6.68 | 1.14 | 0.13 | 108.82 | 11.48 | 0.07 | 0.48 | 7.42 | 33.05 | 54.90 | 12.06 | 41.78 | -23.11 |
|  | B04W | 36.50 | 2.89 | 31.92 | NA | 1.05 | 0.87 | 49.11 | 31.35 | 0.04 | 0.34 | 9.13 | 17.48 | 68.39 | 14.13 | 31.01 | -21.86 |
|  | B04S | 35.00 | 19.30 | 31.40 | 5.90 | 2.30 | 0.17 | 67.40 | 12.80 | 0.04 | 0.30 | 8.90 | 33.24 | 55.07 | 11.70 | 41.87 | -22.82 |
|  | B07S | 65.00 | 8.70 | 32.10 | 7.20 | 2.10 | 0.19 | 94.40 | 9.90 | 0.07 | 0.42 | 7.20 | 32.74 | 51.59 | 15.70 | 34.38 | -22.57 |
|  | B10S | 77.00 | 8.37 | 32.37 | 7.88 | 2.26 | 0.18 | 150.15 | 4.20 | 0.07 | 0.48 | 6.86 | 50.83 | 35.17 | 14.01 | 65.10 | -23.04 |
|  | A3_1W | 18.78 | 8.00 | 28.00 | 13.20 | 0.75 | 0.14 | 19.88 | 4.17 | 0.07 | 0.46 | 8.28 | 11.49 | 69.24 | 19.27 | 16.65 | -23.44 |
|  | A3_1S | 18.00 | 21.00 | 29.00 | 3.18 | NA | NA | NA | NA | 0.08 | 0.58 | 8.87 | 11.22 | 67.39 | 21.39 | 12.35 | -23.64 |
|  | A8_1W | 22.21 | 11.00 | 28.00 | 9.48 | 2.13 | 0.32 | 83.63 | 43.70 | 0.06 | 0.40 | 8.15 | 7.72 | 72.99 | 19.29 | 15.40 | -23.39 |
| CE | A8_1S | 22.00 | 23.00 | 31.00 | 4.55 | NA | NA | NA | NA | 0.06 | 0.40 | 8.00 | 8.62 | 71.21 | 20.19 | 14.00 | -23.06 |
|  | DH11S | 31.00 | 22.20 | 33.10 | 2.40 | NA | NA | NA | NA | 0.11 | 0.95 | 8.30 | 64.51 | 26.44 | 9.08 | 112.50 | NA |
|  | DH25S | 30.00 | 24.20 | 28.80 | 3.60 | NA | NA | NA | NA | 0.22 | 1.22 | 5.40 | 6.95 | 73.03 | 20.04 | 14.00 | NA |
|  | A2_8S | 44.00 | 18.00 | 31.00 | 4.51 | NA | NA | NA | NA | 0.03 | 0.20 | 8.83 | 80.28 | 14.71 | 5.02 | 189.00 | -21.46 |
|  | A8_5W | 67.88 | 13.50 | 34.30 | 12.14 | 0.50 | 0.20 | 6.87 | 3.92 | 0.05 | 0.31 | 7.09 | 54.34 | 30.58 | 15.10 | 74.80 | -21.55 |
| ECS | A8_6S | 62.00 | 22.00 | 32.00 | 4.69 | 3.92 | 0.25 | 152.23 | 32.98 | 0.04 | 0.28 | 7.64 | 65.67 | 23.77 | 10.57 | 102.50 | -21.28 |
|  | P03S | 52.00 | 23.40 | 33.70 | 4.70 | 3.40 | 0.12 | 118.60 | 23.90 | 0.05 | 0.35 | 7.60 | 46.07 | 35.04 | 18.90 | 34.32 | -21.63 |
|  | QT1S | 54.00 | 21.10 | 34.40 | 3.50 | 3.40 | 0.13 | 152.20 | 79.50 | 0.12 | 0.75 | 7.10 | 1.98 | 65.57 | 32.50 | 7.12 | -22.20 |
|  | QT3S | 79.00 | 19.60 | 34.50 | 5.30 | 2.00 | 0.51 | 83.10 | 7.90 | 0.09 | 0.48 | 6.50 | 24.93 | 49.76 | 25.30 | 10.75 | -21.83 |

**Reference**

1. Jorgensen SL, Hannisdal B, Lanzén A, Baumberger T, Flesland K, Fonsecaet R, et al. Correlating microbial community profiles with geochemical data in highly stratified sediments from the Arctic Mid-Ocean Ridge. Proc Natl Acad Sci USA. 2012;109:E2846-E2855.

2. Liu J, Yu S, Zhao M, He B, Zhang X-H. Shifts in archaeaplankton community structure along ecological gradients of Pearl Estuary. FEMS Microbiol Ecol. 2014;90:424-435.
